# Periconceptional biomarkers for maternal obesity: a systematic review

**DOI:** 10.1007/s11154-022-09762-5

**Published:** 2022-12-15

**Authors:** Batoul Hojeij, Melek Rousian, Kevin D. Sinclair, Andras Dinnyes, Régine P. M. Steegers-Theunissen, Sam Schoenmakers

**Affiliations:** 1 Department of Obstetrics and Gynecology, Erasmus MC, University Medical Center, Rotterdam, 3015GD The Netherlands; 2 School of Biosciences, Sutton Bonnington Campus, University of Nottingham, Leicestershire, LE12 6HD UK; 3 BioTalentum Ltd., Godollo, 2100 Hungary; 4 Department of Cell Biology and Molecular Medicine, University of Szeged, Szeged, 6720 Hungary; 5 Department of Physiology and Animal Health, Institute of Physiology and Animal Nutrition, Hungarian University of Agriculture and Life Sciences, Godollo, 2100 Hungary

**Keywords:** Obesity, Body mass index, Periconceptional period, Biomarker

## Abstract

Periconceptional maternal obesity is linked to adverse maternal and neonatal outcomes. Identifying periconceptional biomarkers of pathways affected by maternal obesity can unravel pathophysiologic mechanisms and identify individuals at risk of adverse clinical outcomes. The literature was systematically reviewed to identify periconceptional biomarkers of the endocrine, inflammatory and one-carbon metabolic pathways influenced by maternal obesity. A search was conducted in Embase, Ovid Medline All, Web of Science Core Collection and Cochrane Central Register of Controlled Trials databases, complemented by manual search in PubMed until December 31^st^, 2020. Eligible studies were those that measured biomarker(s) in relation to maternal obesity, overweight/obesity or body mass index (BMI) during the periconceptional period (14 weeks preconception until 14 weeks post conception). The ErasmusAGE score was used to assess the quality of included studies. Fifty-one articles were included that evaluated over 40 biomarkers. Endocrine biomarkers associated with maternal obesity included leptin, insulin, thyroid stimulating hormone, adiponectin, progesterone, free T4 and human chorionic gonadotropin. C-reactive protein was associated with obesity as part of the inflammatory pathway, while the associated one-carbon metabolism biomarkers were folate and vitamin B12. BMI was positively associated with leptin, C-reactive protein and insulin resistance, and negatively associated with Free T4, progesterone and human chorionic gonadotropin. Concerning the remaining studied biomarkers, strong conclusions could not be established due to limited or contradictory data. Future research should focus on determining the predictive value of the optimal set of biomarkers for their use in clinical settings. The most promising biomarkers include leptin, adiponectin, human chorionic gonadotropin, insulin, progesterone and CRP.

**Supplementary Information:**

The online version contains supplementary material available at 10.1007/s11154-022-09762-5.

## Introduction

The global prevalence of obesity almost tripled since 1975, affecting 15% of adult women worldwide [World Health Organization (WHO) 2016] [1]. The rise in this epidemic is alarming for its association with increased reproductive and pregnancy complications [2, 3]. These complications can originate during the periconceptional period (defined as the period from 14 weeks prior to, until 10 weeks following, conception) during which gametogenesis, fertilization, implantation, embryogenesis and placentation take place [4, 5]. From a life-course perspective, maternal obesity impacts the health of the woman and her offspring commencing from the periconceptional period, with the effects persisting into adulthood [4, 6].

Maternal obesity leads to impaired oogenesis, infertility and anovulation [7–10]. In utero, maternal obesity is linked to production of blastocysts with fewer cells, accelerated preimplantation embryonic development, decreased post-implantation embryonic and fetal growth trajectories, and impaired fetal cardiac function [11–16]. Furthermore, obesity continues to pose risks throughout pregnancy such as that for miscarriage, gestational diabetes mellitus (GDM), preeclampsia and delivery complications [17–19]. This, in turn, increases the risk of adverse birth outcomes, neural tube and congenital heart defects in offspring of obese women [20–26].

Unraveling the pathophysiologic mechanisms can aid in understanding the link between maternal obesity and adverse clinical outcomes. Obesity disrupts the endocrine and inflammatory pathways at both systemic and local levels which leads to, or is a consequence of, perturbations in metabolic processes such as one-carbon metabolism [27, 28]. The disruptions can be identified clinically using biomarkers for early diagnosis, for detection and prevention of adverse clinical outcomes [29]. These biomarkers could potentially be used as screening tools to identify population at risk, and to predict outcomes for the mother and offspring. However, the applicability of these biomarkers in a clinical setting is limited and requires more information, particularly among the obese population [30]. For example, it is generally known that low folate levels increase the risk of neural tube defects in offspring, while first trimester inflammatory cytokines are associated with increased risk of preterm birth among obese women [31–33]. Furthermore, endocrine and inflammatory pathways are involved in the manifestation of obesity-related pregnancy complications such as GDM and preeclampsia [34–37].

Identification of biomarkers of the endocrine, inflammatory and one-carbon metabolic pathways affected by maternal obesity during the periconceptional period can aid in our understanding of the pathophysiologic basis of adverse clinical outcomes to be used as an early detection marker of patients at risk [4]. Therefore, the aim of this review is to identify how maternal obesity impacts the different types of biomarkers of the endocrine, inflammatory and one-carbon metabolic pathways during the periconceptional period.

## Methods

### Sources

A literature search was performed by a biomedical information specialist (W.B.) specialized in systematic reviews using the databases of Embase, Ovid Medline All, Web of Science Core Collection and Cochrane Central Register of Controlled Trials until December 31^st^, 2020. The keywords used for the search strategy included but were not limited to: obesity, maternal obesity, pregnancy, preconception, periconception, first trimester, biomarker, endocrinology, leptin, inflammation, carbon metabolism (Table S1). The Boolean operators used for the search outcome were “AND”, “OR” and “NEAR”. In addition, the database of PubMed was manually searched to identify relevant articles. The review was structured in accordance to the Preferred Reporting Items for Systematic Review and Meta-Analyses (PRISMA) guidelines. A protocol of this systematic review was designed and registered under the PROSPERO international prospective registry of systematic reviews (2020: CRD42021240883).

### Eligibility

All types of observational human studies that associated maternal obesity with a certain biomarker measured during the periconceptional period were eligible for inclusion. Biomarkers of the endocrine, inflammatory and one-carbon metabolic pathways were all considered, with no limitation on the source of samples. In addition, eligible studies had to include biomarkers studied in population with obesity or overweight/obesity, or in relation to BMI. Inclusion and exclusion criteria for this systematic review are shown in Table 1.

**Table 1:** Inclusion and exclusion criteria

Criteria	Inclusion	Exclusion	Reason for exclusion
Study design	- Observational	- Intervention	The systematic review is not focused on the impact of intervention on biomarker level
Publication type	- Research papers published in full text	- Non-English papers - No full text available - Reviews - Case reports - Abstracts	Not relevant
Population	- Women	- Men - Animal studies	The systematic review comprises the female population
Period	- Fourteen weeks preconception period - First trimester of pregnancy	- Pregnant women in second and third trimester of pregnancy - Pregnant women with unreported gestational age - Non-pregnant women at any age but not in the 14 weeks preconception period	The time frame of the systematic review encompasses the periconceptional period and additional part of the first trimester of pregnancy
Indication	- Population includes obese women	- Population does not include obese women - Women with identified pregnancy related complications - Women with polycystic ovary syndrome - Women with identified acute and chronic diseases	Population does not include a group of obese or healthy obese women
Outcome	- Endocrine, inflammatory, and one-carbon metabolism biomarkers	- No biomarker measured - No obesity related biomarker outcome - Biomarkers not related to the Endocrine, inflammatory, and one-carbon metabolic pathways	The systematic review is focused on the biomarkers of endocrine, inflammatory, and one-carbon metabolic pathways

Since the aim of this systematic review was to encompass the periconceptional period and the first trimester of pregnancy, articles with a time frame spanning 14 weeks preconception up and until 14 weeks of gestation were considered eligible (we extended the post-conception period from 10 to 14 weeks to include the first trimester of pregnancy).

### Selection strategy

An abstract – title evaluation was performed by two independent reviewers (B.H. and S.S.) on all publications from the search. When both reviewers did not agree on the inclusion of certain articles, a third reviewer (M.R.) repeated the evaluation of the articles for a final decision.

Thereafter, a full text review and data extraction was performed for the selected publications. Data extraction included year of publication, country, study design, detailed sample size, age, BMI, biomarker, biomarker class, gestational age when biomarker and BMI were assessed, biomarker source, statistical analysis and adjustments, results and conclusion.

The systematic review comprised the population of obese women with different fertility status. Obesity was classified based on criteria indicated by the authors of each study, or according to the WHO classification of BMI ≥ 30 kg/m^2^ when no criteria were mentioned. Studies including outcomes related to BMI or combined overweight/obesity were included if the population included obese women identified from the population BMI of  ≥ 30 kg/m^2^ or if the authors indicated N number of obese women, though not studied separately.

### Quality score assessment and risk of bias

The ErasmusAGE score was used to assess the quality of studies included in the systematic review (Table S2) [38]. This tool is based on previously published scoring systems and is applicable for intervention and observational studies, as well as for systematic reviews and meta-analysis [38]. The quality score is based on 5 items covering the study design and size, exposure and outcome, and adjustments. The parameters for these items were adapted, based on the literature and discussion with other researchers, as relevant for the review. The allocated scores for each item were: study design (0 = cross-sectional study, 1 = longitudinal study, 2 = intervention study), study size N (small: 0 =  < 100, intermediate: 1 = 100 to 500, large: 2 =  > 500 participants), exposure measurement method (0 = not reported/inadequate, 1 = moderate quality, 2 = adequate), outcome measurement method (0 = not reported/inadequate, 1 = moderate quality, 2 = adequate) and adjustments in analysis (0 = no adjustments, 1 = controlled for age, 2 = controlled for additional confounders) (Table S2). The score ranges from 0 to 10 and was considered as low (ErasmusAGE score ≤ 5) or high (ErasmusAGE score ≥ 6) for each study.

## Results

### Study selection

**Fig. 1:**
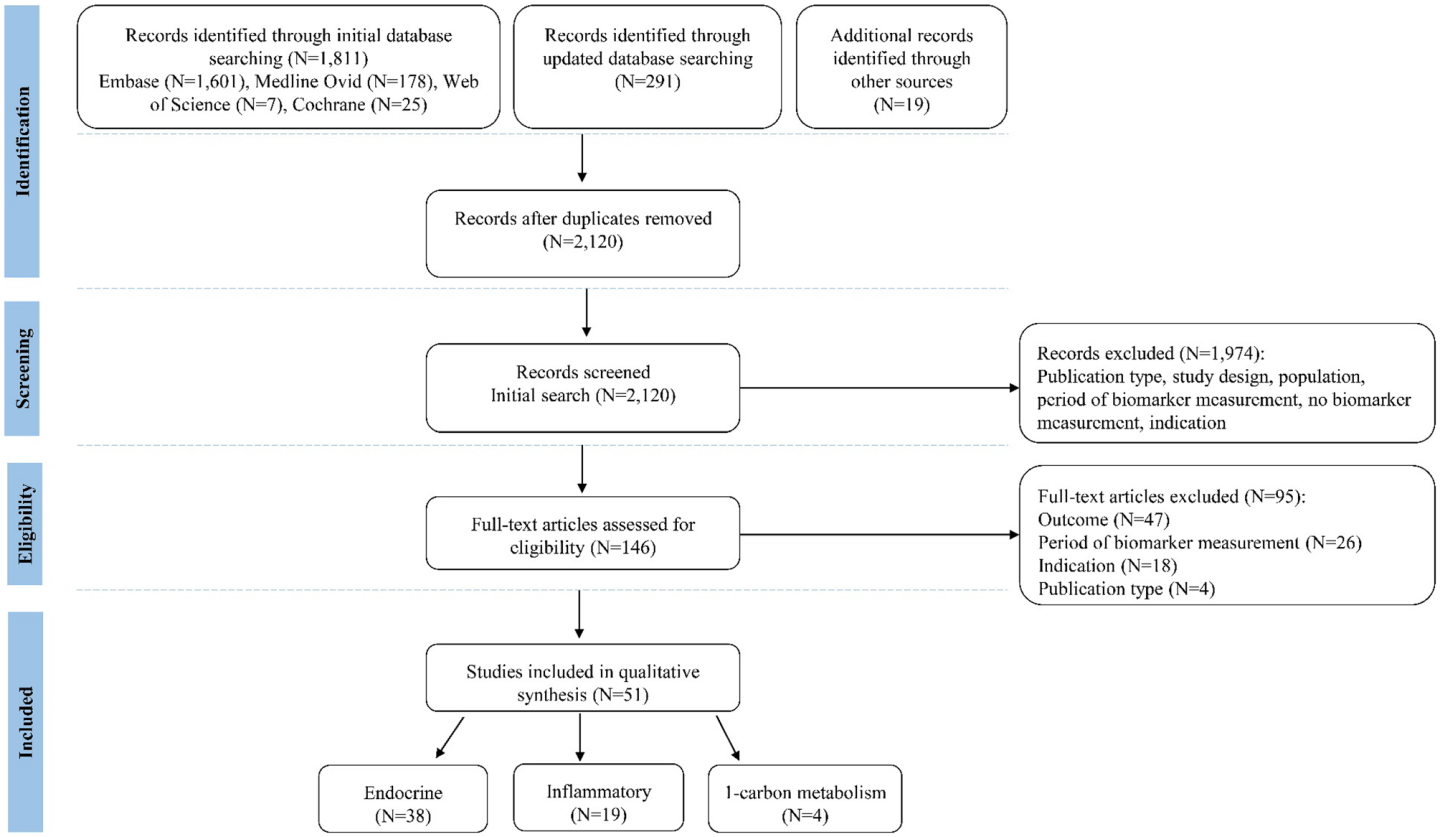
PRISMA flow chart of the systematic review

The flowchart depicted in Fig. 1 summarizes the process of literature screening and study selection*.* The initial and updated electronic search of the databases resulted in 2,102 records (including 1 identified as duplicate) and the manual search resulted in 19 additional records. A total of 1,974 articles were excluded after title-abstract screening for the eligibility criteria, leaving 146 articles for full text assessment. Eventually, 51 articles were left for analysis in this systematic review.

### Study characteristics

Table S3 summarizes the general characteristics and ErasmusAGE quality score of the selected studies. The included studies consisted of prospective (N = 28) and retrospective (N = 3) cohort, cross-sectional (N = 14) and case–control (N = 6) studies. Most studies based their obesity classification according to WHO criteria of BMI ≥ 30 kg/m^2^ (N = 27), or BMI ≥ 25 kg/m^2^ based on BMI references for the Asian population (N = 2). Sources for biomarker sampling included serum, plasma, follicular fluid, placental tissue, red blood cells, urine and oocytes.

### Quality of studies

**Fig. 2:**
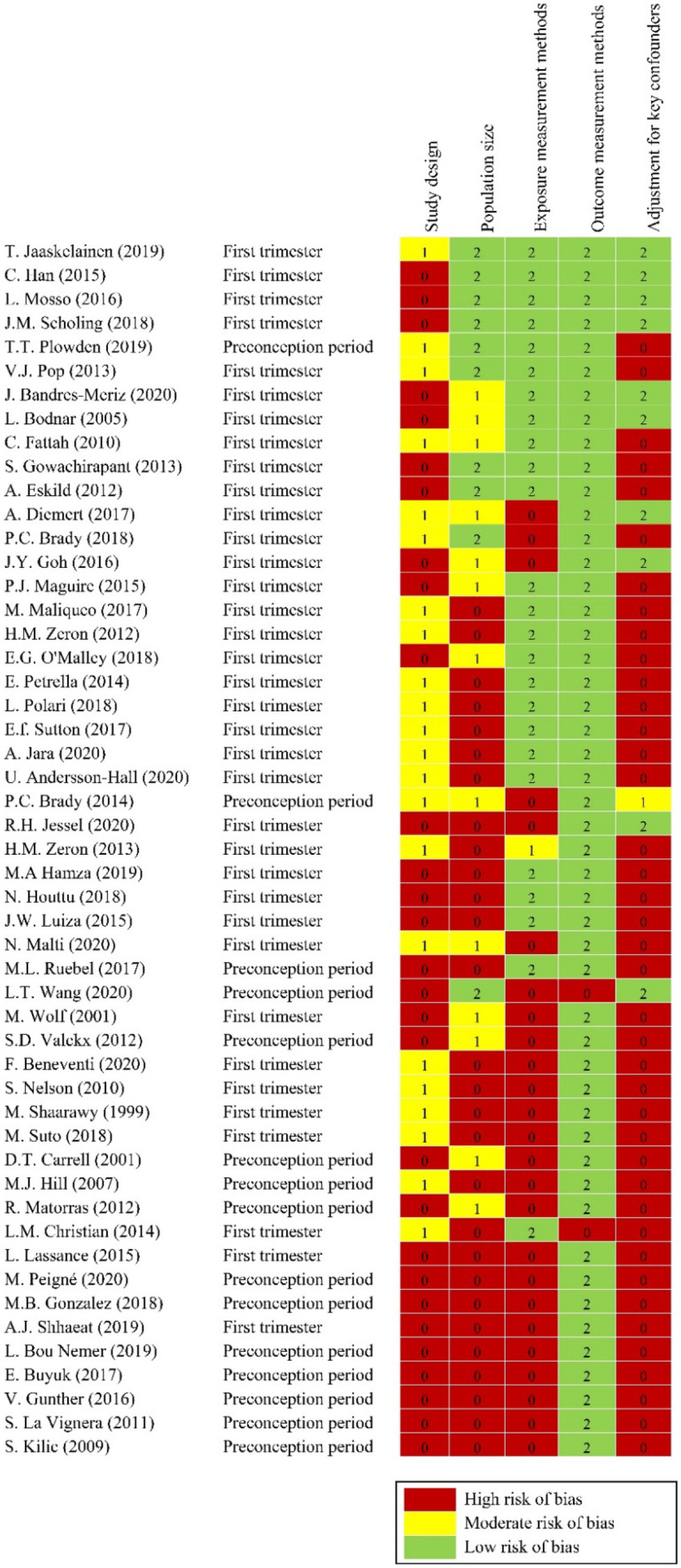
Quality score assessment and risk of bias for all studies included in this systematic review

Details on the quality score and risk of bias assessment for each study are provided in Fig. 2. The quality score of the studies ranged between 2 to 9 (mean ErasusAGE score 4.4 out of 10.0) (Fig. S1). Seventy-six percent of studies were of low quality (N = 39) and 24% were of high quality (N = 12). Fifty-five percent of studies had small sample size (N = 28), 49% did not specify the tool for anthropometrics screening (N = 25), 4% did not specify the analysis tool for biomarker measurement (N = 2), and 78% did not adjust for confounding factors (N = 40). In studies with adjustments for confounding factors (N = 11), the important confounders considered were maternal age (N = 11), gestational age (N = 7), smoking (N = 7) and gravidity/parity (N = 6). Other confounders were alcohol use, race/ethnicity, socio-economic status, marital status, miscarriage, biomarkers, sex of newborn, day of embryo transfer, stress, nausea, weight gain and conception mode; where each was adjusted in one or two studies.

### Endocrine biomarkers

Endocrine biomarkers are considered secretions released into circulation from different glands in the body, interacting with each other in feedback loops. These biomarkers are involved in regulating various body functions including metabolism, growth, appetite and inflammation [39]. Tables 2 and 3 summarize associations between maternal obesity and the endocrine biomarkers for the preconception period and first trimester, respectively.

**Table 2:** Summary of studies examining the association between maternal obesity and biomarkers of the endocrine, inflammatory and one-carbon metabolic pathways during the preconception period sorted according to ErasmusAGE quality score (QS) and biomarker pathway

Author, year	Country	N_Total_, N_Obesity_	Maternal age (years)	BMI criteria (kg/m^2^)	Biomarker	Source	Main findings	QS	Pathw
Plowden et al (2019) [40]	USA	1,053	28.9 ± 4.7	n/a	Leptin	S	*Incr. BMI* *↑* Leptin (r = 0.8) *Incr. waist circumference* *↑* Leptin (r = 0.8)	7	EC
Brady et al (2014) [41]	USA	229 (13)	27.1 ± 4.0	Obesity ≥ 30.0 Overweight 25–29.9 Normal < 25.0	Progesterone	S	*Obesity vs normal weight* ↓ Progesterone (Median (IQR): 16.0 (10.0–50.0) vs 25.0 (6.0–16.0) ng/ml) *BMI* Predictor for progesterone <20 ng/ml	5	EC
Ruebel et al (2017) [42]	USA	24 (11^a^)	Overweight /obesity 32.1 ± 1.5 Normal 31.6 ± 0.9	Overweight /obesity > 25.0 Normal 18.5–24.9	Estradiol, insulin, FSH leptin, LH, CCL2, CRP, IL-6, TNF-α, genes	S, FF, oocyte	*Overweight/obesity vs normal weight* ↑ S HOMA-IR, leptin, CCL2 (Mean ± SD: 1.8 ± 0.3 vs 0.7 ± 0.1, p = 0.002; 27.2 ± 6.6 vs 5.3 ± 1.0 ng/ml, p < 0.003; 403.6 ± 51.0 vs 238.6 ± 22.4 pg/ml, p = 0.005, respectively) ↑ FF leptin, CRP (Mean ± SEM: 28.0 ± 2.0 vs 12.6 ± 6.0 ng/ml, p < 0.05; 5.0 ± 2.0 vs 2.0 ± 0.5 μg/ml, p < 0.05) Ten genes involved in chemokine and cytokine pathways differentially regulated (p = 0.007 and p = 0.011, respectively) *Incr. BMI* ↑ CXCL3, IL-34 gene expression (R = 0.6, p = 0.04) *Incr. fat mass percent* ↑ FF TNF-α (R = 0.7, p = 0.001) *No effect* Obesity on S estradiol, FSH, insulin, LH, CRP, IL-6, TNF-α, and FF insulin, CCL2, TNF-α	4	EC /Inf
Wang et al (2020) [43]	China	2,319 (848)	Group I < 35.0 Group II > 35.0	Obesity 25.0–35.1 Overweight 23.0–25.0 Normal 8.5–23.0	Estradiol, LH, progesterone	S	*Obesity vs normal weight* Natural cycle (group I): ↓ estradiol, LH (Median (IQR): 927.3 (696.1–1201.0) vs 1105.5 (808.8–1426.8), p < 0.05; 25.3 (14.4–43.1) vs 34.3 (19.3–54.5), p < 0.05, respectively) Ovulation induction cycle (group I): ↓ estradiol, LH, progesterone (Median (IQR): 933.5 (525.7–1607.8) vs 1538.0 (827.9–2828.5); 10.70 (7.0–19.0) vs 12.8 (8.1–23.3); 2.1 (1.5–2.9) vs 2.4 (1.7–3.2), ps < 0.05, respectively) *Incr. BMI* ↓ Estradiol, LH, progesterone (r = -0.2, p < 0.001; r = -0.1, p = 0.001; r = -0.1, p < 0.001, respectively) *No effect* Group I (natural cycle): obesity on progesterone Group II (natural and ovulation cycles): obesity on estradiol, LH, progesterone	4	EC
Valckx et al (2012) [44]	Belgium	106 (20)	Obesity 35.0 ± 6.1 Overweight 32.0 ± 4.6 Normal 34.0 ± 4.7	Obesity ≥ 30.0 Overweight 25–29.9 Normal 18.5–24.9	Insulin, CRP, IGF-1	S, FF	*Obesity vs normal weight* ↑ S insulin, CRP, IGF-1 (Mean ± SD: 16.0 ± 9.1 vs 9.0 ± 3.8 mUI/l, p < 0.01; 4.0 ± 3.6 vs 2.0 ± 3.3 mg/l, p < 0.01; 150.0 ± 48.3 vs 185.0 ± 59.7 ng/ml, p = 0.03, respectively) ↑ FF insulin, CRP, IGF-1 (Mean ± SD: 12.0 ± 6.7 vs 8.0 ± 5.3 mUI/l, p < 0.01; 2.0 ± 1.8 vs 2 ± 2.6 mg/l, p < 0.01; 101.0 ± 39.1 vs 128.0 ± 42.4 ng/ml, p = 0.04, respectively) *Incr. BMI* ↓ QUICKI, rQUICKI (ps < 0.01)	3	EC / Inf
Hill et al (2007) [45]	Hawaii	10	33.7 ± 2.8	n/a	Leptin	S, FF	*Incr. BMI* ↑ S and FF leptin (r = 0.8, p < 0.01)	3	EC
Carrell et al (2001) [46]	USA	247 (34)	n/a	High > 30.0 Medium 20.0 < BMI < 30.0 Low < 20.0	Estradiol, hCG	FF	*Obesity vs medium BMI* ↓ hCG (Mean ± SD: 73.6 ± 8.8 vs 121.4 ± 6.0 mIU/ml, p < 0.001) *Obesity vs low BMI* ↓ hCG (Mean ± SD: 73.6 ± 8.8 vs 153.2 ± 11.2 mIU/ml, p < 0.001) *Incr. BMI* ↓ hCG (r = -0.4, p < 0.001) *No effect* Obesity on S estradiol	3	EC
Matorras et al (2012) [47]	Spain	473	34.7 ± 2.9	BMI < 20.0 BMI 20.0–25.0 BMI 25.0–30.0 BMI > 30.0	hCG	P	*Incr. BMI* ↓ hCG (r = -0.4, p < 0.05)	3	EC
Peigné et al (2020) [48]	France	37 (16)	Obesity 31.5 Normal 32.0	Obesity ≥ 30.0 Normal ≤ 25.0	AMH, ASD, estradiol, insulin, LH, FSH, SHBG, testosterone	S	*Obesity vs normal weight* ↓ API, SHBG (34.6 vs 39.0%, p = 0.015; median (5^th^-90^th^ percentile): 36.3 (17.2–65.8) vs 70.4 (40.7–103.8) pmol/l, p < 0.001, respectively) *No effect* Obesity on total AMH, ASD, estradiol, LH, FSH, insulin, proAMH, testosterone	2	EC
Buyuk et al (2017) [49]	USA	S 39 (10) FF 40 (13)	27–43	Obesity ≥ 30.0 Overweight 25.0–29.9 Normal < 25.0	eotaxin, estradiol FSH, CRP, EGF, IL-1α, IL-1β, IL-2, IL-4, IL-6, IL-8, IL-10, GM-CSF, MCP-1, TNF-α	S, FF	*Obesity vs normal weight* ↓ S FSH (Mean ± SD: 6.8 ± 1.7 vs 11.4 ± 4.5 IU/L, p = 0.005) ↑ S CRP, MCP-1 (Mean ± SD: 1586.2 ± 816.5 vs 585.2 ± 321.8 ng/mL, p = 0.0003; 921.1 ± 308.5 vs 558.0 ± 252.7 pg/mL, p = 0.0002, respectively) ↑ FF MCP-1(Mean ± SD: 821.0 ± 403.0 vs 444.0 ± 210.0 pg/ml, p = 0.02) *Incr. BMI* ↑ S CRP, MCP-1, G-CSF and (β = 0.4, p = 0.008; β = 0.4, p = 0.03; β = 0.4, p = 0.03, respectively) ↑ FF MCP-1 (β = 0.5, p = 0.002) *No effect* Obesity on S eotaxin, estradiol, EGF, G-CSF, IL-1α, IL-1β, IL-2, IL-4, IL-6, IL-8, IL-10, TNF-α, and FF estradiol, FSH	2	EC /Inf
Bou Nemer et al (2019) [50]	USA	25 (5)	35.0–45.0	Obesity ≥ 30.0 Overweight 25.0–29.9 Normal 18.5–24.9 Non-obese < 30.0	adiponectin, C-peptide, ghrelin, glucagon, GLP-1, Insulin, leptin, resistin, visfatin, methionine	FF	*Obesity vs non-obese* ↑ C-peptide, glucagon, GLP-1, insulin, leptin (Mean ± SD: 86.0 ± 12.6 vs 56.5 ± 4.9 ng/dl, p = 0.0001; 32.9 ± 6.3 vs 25.3 ± 2.6 pg/ml, p = 0.0008; 43.3 ± 4.8 vs 31.4 ± 1.2 pg/ml, p = 0.0018; 398.0 ± 150.2 vs 96.4 ± 17.5 pg/ml, p = 0.0008; 12.8 ± 1.4 vs 3.6 ± 0.8 ng/ml, p = 0.0001, respectively) *Obesity vs normal* weight ↑ Methionine (p < 0.05) *Incr. BMI* ↑ Methionine (p < 0.05) *No effect* Obesity on adiponectin, ghrelin, resistin *No assoc* BMI with visfatin	2	EC / Inf / 1-CM
Gonzalez et al (2018) [51]	Australia	45–48 (15–16)	n/a	Obesity > 30.0 Overweight 25.0–29.9 Normal n/a	Adiponectin, leptin, CRP, IL-6, IL-10, MCP-1, sICAM-1, TNF-α	FF	*Incr. BMI* ↑ Leptin and CRP (β = 0.1, p < 0.001) ↑ 1 kg/m^2^ assoc with ↑ 14% CRP *No assoc* BMI with adiponectin, IL-6, IL-10, MCP-1, sICAM-1, TNF-α	2	EC /Inf
Kilic et al (2009) [52]	Turkey	82 (24)	n/a	Obesity ≥ 30.0 Overweight 25.0- 30.0 Normal < 25.0	LH, FSH, IL-18	S, FF	*No effect* Obesity on S and FF IL-18, LH, FSH	2	EC /Inf
Gunther et al (2016) [53]	Germany	90 (5)	25.0–43.0	BMI < 20.0 BMI 20.0–25.0 BMI 25.0–30.0 BMI > 30.0	IL-18	S, FF	*Incr. BMI* ↑ S IL-18 with BMI class (p = 0.027) *No assoc* BMI with FF IL-18	2	Inf
La Vignera et al (2011) [54]	Italy	40 (16)	34.0	Obesity (II) 35.0–39.9 Obesity 30.0–34.9 Overweight 25.0–29.9 Normal 18.5–24.9	IL-6, IL-8, TNF-α	FF	*Obesity II vs normal weight* ↑ IL-6, TNF-α (Mean ± SD: 23.1 ± 0.8 vs 14.2 ± 0.6 pg/ml, p < 0.05; 29.8 ± 2.5 vs 15.2 ± 0.6 pg/ml, p < 0.05, respectively) *Obesity vs normal weight* ↑ IL-6 (Mean ± SD: 21.1 ± 1.9 vs 14.2 ± 0.6 pg/ml, p < 0.05) *No effect* Obesity on IL-8	2	Inf

*1-CM* 1-carbon metabolism, *AMH* anti mullerian hormone, *API* anti mullerian hormone prohormone index, *ASD* androstenedione, *assoc* association, *b/w* between, *CCL2* chemokine (C–C motif) ligand 2, *CXCL3* chemokine (C-X-C motif) ligand 3, *EC* endocrine, *EGF* epidermal growth factor, *FF* follicular fluid, *FSH* follicle stimulating hormone, *GIP* gastric inhibitory polypepetide, *GM-CSF* granulocyte macrophage-colony stimulating factor, *hCG* human ghorionic gonadotropin, *IGF-1* insulin-like growth factor 1, *IL* interleukin, *Incr.* increased, *Inf* inflammatory, *LH* luteinizing hormone, *MCP-1* monocyte chemotactic protein-1, *n/a* not available, *pathw* pathway, *IQR* interquartile range, *QS* quality score, *S* serum, *SEM* standard error mean, *SD* standard deviation, *SHBG* sex hormone-binding globulin, *sOB-R* soluble leptin receptor, *TNF* tumor necrosis factor, *P* plasma

(↑) Higher

(↓) Lower

**Table 3:** Summary of studies examining the association between maternal obesity and biomarkers of the endocrine, inflammatory and one-carbon metabolic pathways during the first trimester of pregnancy sorted according to ErasmusAGE quality score (QS) and biomarker pathway

Author, year	Country	N_Total_, N_obesity_	Maternal age (years)	BMI criteria (kg/m^2^)	Biomarker	Source	Main findings	QS	Pathw
Han et al (2015) [55]	China	6,303 (90)	19.0–40.0	Obesity ≥ 30.0 Overweight 25.0–29.9 Normal 18.5–24.9	FT4 and TSH	S	*Obesity vs overweight* ↑ TSH (Median (2.5^th^–97.5^th^ percentile): 2.5 (0.6–13.8) vs 2.1 (0.4–6.8) mIU/l, p < 0.001) ↑ TSH > 5.2 mIU/l (OR 2.4, 95% CI 1.2, 5.0) ↓ FT4 (Median (2.5^th^–97.5^th^ percentile): 14.7 (10.3–18.8) vs 15.3 (11.9–19.7) pmol/l, p < 0.001) *Incr. BMI* 1 kg/m^2^ assoc with ↓ 0.12 pmol/l FT4 (p < 0.05)	8	EC
Mosso et al (2016) [56]	Chile	720 (145)	25.4 ± 6.6	Obesity ≥ 30.0 Normal 20.0–24.9	FT4, TSH, TT4	S	*Obesity vs normal weight* ↑ TSH (Median: 2.3 vs 2.0 mIU/l, p = 0.036) ↓ FT4 (Median: 13.9 vs 14.8 pmol/l, p < 0.01) *Incr. BMI* ↑ TSH (R^2^ = 0.009, p = 0.04) ↓ FT4 (R^2^ = -0.09, p < 0.001) ↓ TT4 with BMI class (p = 0.002)	8	EC
Pop et al (2013) [57]	NL	1,035 (160)	30.5 ± 3.6	Obesity (III) > 40.0 Obesity (II) 35.0–40.0 Obesity (I) 30.0–35.0 Pre-obesity 25.0–30.0 Normal 18.5–25.0 Underweight > 18.5	FT4 and TSH	n/a	*Incr. BMI* ↓FT4 (r = -0.14, p < 0.001) Diff. in FT4 and TSH b/w BMI classes (p < 0.001 and p = 0.02, respectively) *No assoc* BMI with TSH	7	EC
Bandres-Meriz et al (2020) [58]	Austria	323 (24)	29.5 ± 7.0	n/a	C-peptide, insulin, leptin	S	*Obesity vs normal/ underweight* ↑ C-peptide, leptin (Median (IQR): 545.9 (431.7–680.8) vs 329.1 (258.0–422.1) pmol/l, p < 0.001; 20.6 (16.4–29.1) vs 8.0 (4.0–11.7) ng/ml, p < 0.001, respectively) ↓ ISOHOMA (Median (IQR): 0.5 (0.4–0.7) vs 0.8 (0.6–1.1), p < 0.001) *Incr. BMI* ↑ Leptin (r = 0.5, p < 0.001)	7	EC
Diemert et al (2017) [59]	Germany	220 (18)	32.0 ± 3.7	Obesity > 30.0	Progesterone	S	*Incr. BMI* ↓ Progesterone (β = -1.1, p < 0.0001)	6	EC
Eskild et al (2012) [60]	Norway	2,626 (231)	32.7	BMI < 20.0 BMI 20.0–25.0 BMI 25.0–30.0 BMI 30.0–35.0 BMI ≥ 35.0	hCG	S	*BMI* ≥ *35.0 vs BMI* < *20.0* Singleton prg.: ↓ hCG (Mean ± SD: 97.0 ± 51.0 vs 136.0 ± 76.0 IU/l, p < 0.05) Multiple prg.: ↓ hCG (Mean ± SD:152.0 ± 68.0 vs 252.0 ± 123.0 IU/l, p < 0.05) *Incr. BMI* Total pop.: ↓ hCG (R^2^ = -0.013, p < 0.0001) Singleton prg.: ↓ hCG (R^2^ = -0.012, p < 0.0001) Multiple prg.: ↓ hCG (R^2^ = -0.03, p < 0.0001)	6	EC
Gowachirapant et al (2014) [61]	Thailand	514 (79)	30.0 ± 5.0	Obesity ≥ 25.0 Normal 18.5–22.9	FT4, Tg, TSH	S	*Incr. BMI* ↑ Tg (β = 0.1, p < 0.023) ↓FT4 (β = -0.2, p < 0.001) *No assoc* BMI not predictor for TSH	6	EC
Fattah et al (2011) [62]	Ireland	100 (17)	28.1 ± 5.1	Obesity > 29.9	Leptin	S	*Incr. BMI* ↑ Leptin (R = 0.5) *Incr. fat mass* ↑ Leptin (R = 0.5, p < 0.0001)	6	EC
Brady et al (2018) [63]	USA	541	Obesity^a^ 34.4 ± 3.9 Non-obesity^a^ 33.3 ± 3.3	Obesity III > 40.0 Obesity II 35.0–39.9 Obesity I 30.0–34.9 Non-obese < 30.0	hCG	S	*Obesity vs non-obese* ↓ hCG (β = -170.38, 95% CI -199.6, -141.2) ↑ hCG < 100 mIU/ml (OR 3.8, 95% CI 1.8, 7.9) *Incr. BMI* ↓ hCG with BMI class (p < 0.0001)	5	EC
Goh et al (2016) [64]	Singapore	194 (14)	29.9 ± 4.0	Obesity ≥ 30.0 Overweight 25.0–29.9 Normal 18.5–24.9	Progesterone	S	*Obesity vs normal weight* ↓ Progetseone (Mean ± SD: 46.9 ± 18.1 vs 65.5 ± 21.6 nmol/l, p = 0.004); ↑ Progesterone < 35 nmol/l (OR 9.1, p = 0.003) *Incr. BMI* ↓ Progesterone with BMI class (p = 0.015)	5	EC
Andersson-Hall et al (2020) [65]	Sweden	49 (19)	Obesity 31.8 ± 3.5 Healthy 31.7 ± 3.4	Obesity ≥ 30.0 Healthy 18.5–24.9	Adiponectin, insulin, leptin, sOB-R	S	*Obesity vs healthy weight* ↑ Free leptin index, HOMA-IR, leptin, leptin/adiponectin (ps ≤ 0.001) ↓ Adiponectin and sOB-R (ps ≤ 0.001) *Incr. fat mass percent* ↑ HOMA-IR (β = 0.38, p = 0.037)	5	EC
Jara et al (2020) [66]	USA	80 (19)	25.6 ± 4.3	Obesity ≥ 30.0 Overweight 25.0–29.9 Normal 18.5–24.9	Adiponectin, leptin	S	*Obesity vs normal weight* ↑ Leptin (p < 0.005) ↓ Adiponectin (p < 0.02) *Incr. BMI* ↑ Leptin (r > 0.55, p < 0.001) ↓ Adiponectin (r < -0.28, p < 0.01)	5	EC
Maliqueo et al (2017) [67]	Chile	71 (36)	24.0	Obesity ≥ 30.0 Normal 20.0–24.9	Adiponectin, ASD, estradiol, estrone, insulin, progesterone, SHBG, testosterone	S	*Obesity vs normal weight* Total pop.: ↑ HOMA-IR, fasting insulin, FAI, testosterone (Median (IQR): 2.4 (2.3–3.2) vs 1.7 (1.2–2.2), p < 0.001; 14.0 (12.6–17.5) vs 10.0 (7.4–12.6) μUI/ml, p < 0.001; 0.5 (0.4–0.7 vs 0.25 (0.24–0.6), p = 0.003; 2.1 (3.1–1.5) vs 1.25 (1.0–3.0) nmol/l, p = 0.01, respectively) ↓ Adiponectin and progesterone (Median (IQR): 8.6 ( 6.3–11.5) vs 11.7 (10.1–13.2) μg/ml, p = 0.001; 7.0 (5.0–9.0) vs 8.0 (7.5–10.0) p = 0.014, respectively) Pregnancies with male fetus: ↓ Progesterone (Median (IQR): 7.0 (5.0–8.5) vs 10.0 (8.5–12.5), p = 0.034) ↑ FAI, testosterone (Median (IQR): 0.7 (0.4–1.4) vs 0.3 (0.2–0.4), p = 0.011; 3.0 (1.9–4.0) vs 1.8 (1.5–2.0) nmol/l, p = 0.023, respectively) *No effect* Obesity on ASD, estradiol, estrone, SHBG	5	EC
Zeron et al (2012) [68]	Mexico	40^b^ (21)	Overweight /obesity 26.1 ± 4.6 Normal 24.8 ± 5.7	Obesity ≥ 30.0 Overweight /obesity ≥ 25.0 Overweight 25.0- 30.0 Normal < 25.0	Leptin	S	*Overweight/obesity vs normal weight* ↑ Leptin (Mean ± SD: 29.6 ± 11.0 vs 45.8 ± 13.1 ng/ml, p < 0.001)	5	EC
Petrella et al (2014) [69]	Italy	22 (12)	Overweight /obesity 32.1 ± 5.4 Normal 25.6 ± 4.5	Overweight /obesity ≥ 25.0 Normal 18.0–25.0	Insulin	S	*Overweight/obesity vs normal weight* ↑ Fasting insulin (Mean ± SD: 17.6 ± 16.5 vs 8.0 ± 5.1 mUI/l, p < 0.01)	5	EC
Houttu et al (2018) [70]	Finland	99 (47)	30.0 ± 5.0	Obesity ≥ 30.0 Overweight 25.0–29.9	Insulin, CRP, GlycA	S	*Obesity vs overweight* ↑ HOMA2-IR, insulin, CRP, GlycA (6.1 vs 4.0 mg/l, p = 0.002; 14.0 vs 9.0 mU/I, p < 0.001; 1.7 vs 1.2, p < 0.001; 1.6 ± 0.2 vs 1.5 ± 0.1 mmol/l, p < 0.001, respectively) *Incr. BMI* ↑ CRP, GlycA (rho = 0.4, p < 0.001; rho = 0.5, p < 0.001, respectively) Explanatory factor for HOMA2-IR (β = 0.072, p < 0.001)	4	EC /Inf
Malti et al (2020) [71]	Algeria	120 (60)	Obesity 31.0 ± 3.0 Normal 28.0 ± 3.0	Obesity ≥ 30.0 Normal 19.0–25.0	leptin and insulin	P	*Obesity vs normal weight* ↑ Insulin, leptin (Mean ± SD: 2.7 ± 0.2 vs 1.2 ± 0.1 ng/ml, p = 0.006; 30.3 ± 2.2 vs 11.5 ± 1.2 ng/ml, p < 0.05, respectively)	4	EC
Luiza et al (2015) [72]	USA	50	Depressed 22.3 ± 3.1 Non-depressed 22.2 ± 3.0	Obesity ≥ 30.0	Cortisol	P, U	*Obesity vs non-obese* Non-depressed pop.: ↓ P cortisol (Median (IQR: 22.0 (19.0–38.0) vs 40.0 (25.0–51.0) ng/ml, p < 0.05) *Incr. BMI* Non-depressed pop.: ↓ P cortisol (r^2^ = -0.29, p < 0.01) *Incr. fat mass and percent* Total pop.: ↓ P cortisol (r^2^ = -0.10, p < 0.05) Non-depressed pop.: ↓ P cortisol (r^2^ = -0.29, p < 0.01; r^2^ = -0.26, p < 0.01, respectively) *No effect* Depressed pop.: obesity on P cortisol *No assoc* Total pop.: BMI with U and P cortisol Depressed pop.: BMI and fat with P cortisol	4	EC
Hamza et al (2019) [73]	Iraq	Prg.^c^ 20 (8^d^)	LP 24.0 ± 3.8 OP 27.6 ± 3.9	Overweight /obesity > 25.0 Normal ≤ 24.9	Betatrophin	n/a	*Overweight/obesity vs normal weight* ↓ Betatrophin (Mean: 2.5 vs 6.1 ng/ml, p < 0.001)	4	EC
Zeron et al (2013) [74]	Mexico	42 (21)	Overweight/obesity 28.0 Normal 21.0	Overweight /obesity ≥ 25.0 Normal < 25.0	Adiponectin and leptin	S	*Overweight/obesity vs normal weight* ↑ Leptin (Median (IQR): 18.3 (11.0–24.9) vs 9.6 (2.3–13.3) ng/ml, p < 0.001) ↓ Adiponectin/leptin (Median (IQR): 4.7 (3.1–7.1) vs 12.0 (4.1–46.2), p < 0.05) *No effect* Obesity on adiponectin	4	EC
Beneventi et al (2019) [35]	Italy	82 (35^d^)	Overweight/obesity 31.0–36.0 Controls 29.7–38.0	Overweight /obesity ≥ 24.9 Normal < 24.9	Leptin and IL-33	S	*Incr. BMI* ↑ Leptin (rho = 0.4, p < 0.001) ↓ IL-33 (rho = -0.4, p < 0.001)	3	EC /Inf
Nelson et al (2010) [75]	UK	60	n/a	n/a	AMH	P	*Incr. BMI* ↓ AMH (r = -0.33, p = 0.01) *Incr. waist circumference* ↓ AMH (r = -0.31, p = 0.018)	3	EC
Shaarawy (1999) [76]	Egypt	11^e^	31 ± 5.1	n/a	Leptin	S	*Incr. BMI* ↑ Leptin (r = 0.89, p < 0.01)	3	EC
Suto et al*.* (2019) [77]	Japan	89 (18^d^)	Overweight/obesity 29.2 ± 4.3 Normal 31.5 ± 5.2 Lean 29.1 ± 6.9	Oveweight /obesity ≥ 25.0 Normal 18.0–25.0 Lean < 18.5	Adiponectin, leptin, resistin, visfatin	S	*Overweight/obesity vs normal weight* ↑ Leptin, visfatin (Mean: 25.0 vs 12.0 ng/ml, p < 0.001; 1.1 vs 0.8 ng/ml, p < 0.05, respectively) ↓ Adiponectin (Mean: 8.0 vs 12.5 μg/ml, p = 0.054) *No effect* Obesity on resistin	3	EC
Lassance et al (2015) [78]	USA	33 (16)	Obesity 24.3 ± 4.9 Normal 25.5 ± 7.0	Obesity > 30.0 Normal < 25.0	Insulin, leptin, placental insulin regulated genes	P, Plac. T	*Obesity vs normal weight* ↑ Insulin, leptin (Mean ± SD: 13.2 ± 4.9 vs 7.0 ± 5.2 μU/mL, p = 0.002; 40.8 ± 20.8 vs 16.5 ± 12.8 ng/ml, p < 0.001, respectively) Insulin treated cells: ↓ genes regulated by insulin (-30x, 87 vs 2875) Untreated cells: 1342 genes differentially expressed (90% down expressed)	2	EC
Jaaskelainen et al (2019) [79]	Finland	2510	n/a	Obesity ≥ 30.0 Overweight 25.0–29.9 Normal < 25.0	CRP	S	*Obesity vs normal weight* ↑ CRP (Mean ± SD: 9.2 ± 7.3 mg/l vs 4.2 ± 7.8 mg/l, p < 0.001) *BMI* Assoc with CRP in PE and non-PE (R^2^ = 0.2, p < 0.001)	9	Inf
Scholing et al (2018) [80]	NL	4243 (211)	30.9 ± 4.9	Obesity ≥ 30.0 Normal 18.5–24.9	CRP, folate, vitamin B12	S	*Obesity vs normal weight* ↑ CRP (Median (IQR): 8.2 (5.0–14.4) vs 2.8 (1.2–5.2) mg/l, p < 0.001) ↓ Folate, vitamin B12 (β = -2.3, p < 0.01; -15%, p < 0.001, respectively) ↑ Folate def., vitamin B12 def. (OR = 2.0, p < 0.01; OR = 2.1, p < 0.001, respectively)	8	Inf /1-CM
Bodnar et al (2005) [81]	USA	220 (36)	PE 25.3 ± 6.1 Controls 25.4 ± 6.1	Obesity ≥ 30.0 Normal 18.5–24.9 Underweight < 18.5	CRP	S	*Obesity vs normal weight* ↑ CRP (Mean: 0.7 vs 0.3 mg/dl, p < 0.05) *BMI* ↑ 5 units assoc with ↑ 46% CRP (95% CI 33, 61)	7	Inf
Maguire et al (2015) [82]	Ireland	146 (22)	18.6–43.7	n/a	CRP	S	*Incr. BMI* ↑ CRP (R^2^ = 0.1, p < 0.001)	5	Inf
Polari et al (2018) [83]	Finland	78 (9)	n/a	Obesity > 30.0 Overweight 25.0–30.0 Normal 18.5–24.9	MCP-1, IL-1β, IL-10	S	*Obesity vs normal weight* ↑ MCP-1 (+ 42%, p < 0.001) *No effect* Obesity on IL-10, IL-1β	5	Inf
Sutton et al (2018) [84]	USA	43 (29)	29.0 ± 5.0	Obesity ≥ 30.0 Overweight 25.0–29.9	FGF21	S	*Incr. BMI* ↑ FGF21 (rho = 0.5, p = 0.008) *Incr. fat mass* ↑ FGF21 (rho = 0.5, p = 0.01)	5	Inf
Wolf et al (2001) [85]	USA	120	29.5	n/a	CRP	S	*Incr. BMI* ↑ CRP (r = 0.4, p < 0.01)	3	Inf
Christian et al (2014) [86]	USA	57 (24)	Obesity 24.3 ± 3.7 Overweight 24.1 ± 3.5 Normal 24.3 ± 3.7	Obesity ≥ 30.0 Overweight 25.0–29.9 Normal 18.5–24.9	CRP, IL-1β, IL-6, IL-8, TNF-α	S	*Obesity vs normal weight* ↑ IL-6 (Mean: 0.3 vs 0.1 log pg/ml, p < 0.04) *No effect* Obesity on CRP (p ≥ 0.05), IL-1β, IL-8, TNF-α	3	Inf
Shhaeat et al (2019) [87]	Iraq	60	25–35	Obesity > 30.0 Healthy n/a	CRP	S	*Obesity vs healthy weight* ↑ CRP (Mean ± SD: 8.7 ± 0.2 vs 5.2 ± 0.1 mg/l, p < 0.05)	2	Inf
O'Malley et al (2018) [88]	Ireland	496 (97)	30.7 ± 5.5	Obesity ≥ 30.0 Normal 18.5–24.9	Folate and vitamin B12	S, P, RBC	*Obesity vs normal weight* ↓ S folate, P vitamin B12 (Median (IQR): 32.0 (20.2) vs 36.2 (16.3) nmol/l, p = 0.02; 203.0 vs 208.0 pmol/l, p = 0.03, respectively) ↑ Vitamin B12 < 148 pmol/l (24.7% vs 15.3%, p = 0.037) *Incr. BMI* ↓ S folate, P vitamin B12 (rho = -0.1, p = 0.03; rho = -0.1, p = 0.026) *No effect* Obesity on RBC folate *No assoc* BMI with RBC folate	5	1-CM
Jessel et al (2020) [89]	USA	24 (11)	Obesity 29.5 ± 1.5 Normal 23.3 ± 1.5	Obesity ≥ 30.0 Normal 18.5–24.9	Folate, MTHF, MVM FR-α, MVM PCFT, MVM RFC	S, Plac. T	*Obesity vs normal weight* ↓ Expression of MVM FR-α, MVM RFC (-17% p = 0.037; -19%, p = 0.026, respectively) ↓ MTHF uptake (-52%, p = 0.016) *No effect* Obesity on S folate and MVM PCFT expression	4	1-CM

*1-CM* 1-carbon metabolism, *AMH* anti mullerian hormone, *assoc* association, *b/w* between, *CRP* C-reactive protein, *def.* deficiency, *diff.* difference, *EC* endocrine, *FGF21* fibroblast growth factor 21, *FR-α* folate receptor α, *GlycA* glycoprotein acetylation, *GA* gestation weeks, *Incr.* increased, *IL* interleukin, *Inf* inflammatory, *LP* lean pregnant, *MCP-1* monocyte chemotactic protein-1, *MTHF* methyl tetrahydrofolate, *MVM* microvillous plasma membrane, *NL* Netherlands, *n/a* not available, *OP* obese pregnant, *P* plasma, *pathw* pathway, *PCFT* proton coupled folate transporter, *PE* preeclemptic, *Plac. T* placental tissue, *pop.* population, *Prg.* pregnant, *IQR* interquartile range, *QS* quality score, *RBC* red blood cells, *RFC* reduced folate carrier, *S* serum, *SD* standard deviation, *SHBG* sex hormone-binding globulin, *sOB-R* soluble leptin receptor, *Tg* thyroglobulin, *TNF* tumor necrosis factor, *TSH* thyroid stimulating hormone, *U* urine

^a^ oocyte age

^b^ N total population: 40 (healthy) and 16 (GDM)

^c^ N total population: 59 (all three trimesters) and 22 (non-pregnant)

^d^ Overweight/obesity

^e^ N total population: 52 (all three trimesters) and 30 (non-pregnant women)

(↑) Higher

(↓) Lower

#### Adipokines

The studied adipokines included leptin, adiponectin, visfatin and resistin and they were analyzed in seventeen different studies (cohort N = 11, cross-sectional N = 4, case–control N = 2) [58, 62, 65–68, 71, 74, 76–78].

##### Leptin

Leptin was analyzed in sixteen different studies (mean ErasmusAGE score 4.1 out of 10.0), three of high quality and thirteen of low quality.

Preconception: Higher levels of follicular fluid and serum leptin were observed in obese and overweight/obese women compared to women of normal weight in two studies [42, 50]. Additionally, BMI was positively correlated with follicular fluid leptin levels in three studies (i.e., higher levels of leptin with increasing BMI) [40, 45, 51].

First trimester: The levels of leptin were higher in obese (five studies) and overweight/obese (three studies) women compared to women of normal weight [58, 65, 66, 68, 71, 74, 77, 78]. Also, BMI positively correlated with maternal leptin levels in five studies [35, 58, 62, 66, 76]. Furthermore, Fattah et al*.* demonstrated a positive correlation between fat mass and leptin levels [62].

##### Adiponectin

Adiponectin was analyzed in seven different studies of low quality (mean ErasmusAGE score 3.7 out of 10.0).

Preconception: No difference in follicular fluid adiponectin levels was observed between obese and non-obese women in one study; another study reported no correlation with BMI [50, 51].

First trimester: Obese women exhibited lower levels of adiponectin compared to women of normal weight in three studies [65–67]. BMI was also negatively correlated with adiponectin levels in one study (i.e., lower levels of adiponectin with increasing BMI) [66]. One study showed that adiponectin levels in overweight/obese women were lower compared to women of normal weight, while no difference was observed in another study [74, 77].

##### Visfatin and resistin

Visfatin and resistin were analyzed in two different studies of low quality (mean ErasmusAGE score 2.5 out of 10.0).

Preconception: In one study the effect of obesity and BMI on resistin and visfatin levels was investigated, but no difference or correlation was found [50].

First trimester: Suto et al*.* reported higher levels of visfatin in overweight/obese women compared to women of normal weight, whereas no difference was observed in resistin levels [77].

#### Thyroid biomarkers

Biomarkers of the thyroid function included free T4 (FT4), thyroid stimulating hormone (TSH), total T4 (TT4) and thyroglobulin (Tg). These were analyzed in four different studies (cohort N = 2, cross-sectional N = 2) [55–57, 61].

##### FT4

FT4 was analyzed in four different studies of high quality (mean ErasmusAGE score 7.3 out of 10.0).

First trimester: In three different studies, FT4 levels were lower in obese compared to non-obese women [55–57]. In three further studies, BMI was negatively associated with FT4 levels (i.e., lower levels of FT4 with increasing BMI) [56, 57, 61]. Han et al*.* illustrated this effect by showing that each 1 kg/m^2^ increase in BMI was associated with a 0.12 pmol/l lower FT4 levels [55].

##### TSH

TSH was analyzed in four different studies of high quality (mean ErasmusAGE score 7.3 out of 10.0).

First trimester: TSH levels were elevated in obese compared to non-obese women in three studies [56, 57]. Also, Han et al*.* showed an association between obesity and increased odds for higher TSH levels [55]. A positive association between BMI and TSH levels was shown in one study, whereas no association was established in two other studies [56, 57, 61].

##### Tg and TT4

First trimester: BMI was positively associated with Tg concentrations in one study (ErasmusAGE score 6.0 out of 10.0), whereas lower TT4 levels were observed with increasing BMI class in another study (ErasmusAGE score 8.0 out of 10.0) [56, 61].

#### Steroids

Steroid analyses were reported in ten studies and included estrogen, progesterone, androestardione, testosterone, and cortisol (cohort N = 6, cross-sectional N = 2, case–control N = 2) [41–43, 46, 48, 49, 59, 64, 67, 72].

##### Estrogen

Estrogen was analyzed in six different studies of low quality (mean ErasmusAGE score 3.3 out of 10.0).

Preconception: No difference was observed in either serum or follicular fluid levels of estradiol between obese women compared to women of normal weight in five studies, with an exception of a subgroup of women aged < 35 years that showed that lower levels were associated with obesity in one study [42, 43, 46, 48, 49]. Additionally, one study showed a negative correlation between BMI and serum estradiol levels [43].

First trimester: In one study, no difference was observed in serum estradiol and estrone levels between obese women compared to women of normal weight [67].

##### Progesterone

Progesterone was analyzed in five different studies (mean ErasmusAGE score 5.0 out of 10.0), one of high quality and four of low quality.

Preconception: In two different studies, obese women undergoing assisted reproductive technology (ART) treatment had lower progesterone levels compared to women of normal weight, whereas in one study, no difference was observed in obese women compared to women of normal weight in natural cycle pregnancies, nor in women aged > 35 years [41, 43].

First trimester: Obese women had lower progesterone levels compared to women of normal weight in two studies [64, 67]. This difference was evident in the total studied population and in pregnancies with male fetuses; as well as by association between obesity and higher odds for lower progesterone levels [64, 67]. Also, a negative association was observed between BMI and progesterone levels in two studies [59, 64].

##### Androstenedione, testosterone, cortisol

Testosterone and androstenedione were analyzed in two different studies of low quality (mean ErasmusAGE score 3.5 out of 10.0), whereas cortisol was analyzed once (ErasmusAGE score 4.0 out of 10.0).

Preconception: No difference in testosterone and androstenedione levels was observed between obese women and women of normal weight in one study [48].

First trimester: One study showed that the levels of testosterone and the free androgen index were higher in obese women compared to women of normal weight in the total study population, and in pregnancies with male fetuses; whereas no difference was observed in androstenedione levels [67]. In a group of depressed and non-depressed women, plasma cortisol levels were negatively associated with fat mass and fat percentage, while no association was evident with BMI [72]. Further subgroup analysis showed that plasma cortisol levels were lower in obese compared to non-obese women (but only for those that were non-depressed), and levels of cortisol associated negatively with BMI, fat mass and fat percentage [72].

#### Gonadotropins

Gonadotropins were analyzed in nine different studies and included follicular stimulating hormone (FSH), luteinizing hormone (LH) and human chorionic gonadotropin (hCG) (cohort N = 3, cross-sectional N = 5, case–control N = 1) [42, 43, 46–49, 52, 60, 63].

##### FSH and LH

FSH and LH were analyzed in four different studies of low quality (mean ErasmusAGE score 2.5 and 3.0 out of 10.0, respectively).

Preconception: Apart from one study that revealed lower levels of serum FSH in obese compared to non-obese women, no differences were observed in four studies in either serum or follicular fluid FSH levels [42, 48, 49, 52]. Similarly, only one study with a subgroup of women aged < 35 years, reported lower levels of serum LH in obese women compared to women of normal weight, whereas no difference was observed in women aged > 35 years. This was similar to the findings of three other studies [42, 43, 48, 52]. Finally, in one study, BMI correlated negatively with serum LH levels [43].

##### HCG

HCG was analyzed in four different studies (mean ErasmusAGE score 4.3 out of 10.0), one of high and three of low quality.

Preconception: Lower follicular fluid hCG levels in obese compared to non-obese women were established in one study, and plasma and follicular fluid levels correlated negatively with BMI in two further studies [46, 47].

First trimester: Serum hCG levels were lower in obese compared to non-obese women, and correlated negatively with BMI in two studies [60, 63].

#### Insulin

Insulin was analyzed in eleven different studies (cohort N = 9, case–control N = 1, cross-sectional N = 1; mean ErasmusAGE score 3.9 out of 10.0), predominantly of low quality (N = 10 out of 11).

Preconception: Two studies showed that serum or follicular fluid levels of insulin were higher in obese women compared to women of normal weight, whereas no difference was observed in either sources in two other studies [42, 44, 48, 50]. The increase in insulin levels was supported by a negative correlation between BMI and insulin sensitivity in one study, and increased insulin resistance in overweight/obese women compared to women of normal weight in another study [42, 44].

First trimester: Higher levels of insulin were observed in obese (four studies) and overweight/obese (one study) women compared to normal weight or overweight women [67, 69–71, 78]. HOMA-IR and HOMA2-IR scores (used to assess insulin resistance) were higher in obese women compared to normal weight or overweight women in three studies; whereas the ISHOMA score, which assesses insulin sensitivity, was lower [58, 65, 67, 70]. Also, insulin sensitivity decreased with increasing BMI in one study; while BMI was a significant explanatory factor for HOMA2-IR in another study [58, 70]. What is more, fat mass correlated positively with HOMA-IR score in one study [65]. Another study on insulin treated placental trophoblasts showed that, among obese women, genes responding to insulin (87 identified) were 30 times less abundant compared to women of normal weight (2,875 genes) [78]. Moreover, in untreated placental cells, 1,342 genes were differentially expressed between obese women compared to women of normal weight; with 90% showing down regulated expression [78].

#### Other endocrine biomarkers

Anti-Müllerian hormone (AMH), C-peptide and sex hormone binding globulin (SHBG) were each analyzed in two different studies of low quality (mean ErasmusAGE score 2.5, 4.5 and 3.5 out of 10.0, respectively), whereas betatrophin, glucagon, glucagon-like peptide-1 (GLP-1), ghrelin, insulin like growth factor-1 (IGF-1), and soluble leptin receptor (sOB-R) were analyzed only once (ErasmusAGE scores ≤ 5.0 out of 10).

Preconception: Serum IGF-1 and follicular fluid IGF-1, glucagon, GLP-1 and C-peptide were analyzed once and showed higher levels in obese women compared to women of normal weight [44, 50]. One other study reported lower serum SHBG levels in obese women compared to women of normal weight [48]. AMH, proAMH and ghrelin were each analyzed once and showed no difference between obese and non-obese women [48, 50].

First trimester: One study analyzed AMH and reported a negative correlation with BMI and waist circumference [75]. Also, levels of sOB-R and betatrophin were each lower in obese and overweight/obese women compared to women of normal weight, respectively [65, 73]. In contrast, obese women had higher levels of C-peptide compared to women of normal weight in one study [58]. No difference was observed in SHBG levels between obese women and women of normal weight in one study [67].

##### Highlight

Obesity alters several endocrine biomarkers throughout the periconceptional period. In particular, leptin and insulin levels are increased, whereas levels of adiponectin, FT4, hCG and progesterone are decreased.

### Inflammatory biomarkers

It is widely accepted that obesity represents a state of chronic inflammation. The inflammatory process is a body defense mechanism against injury or infection triggered by cellular and tissue damage during which various substances are released into circulation. These represent inflammatory biomarkers. These biomarkers can be used as a measure of health status and disease progression. Tables 2 and 3 summarize associations between maternal obesity and inflammatory biomarkers for the preconception period and first trimester, respectively.

#### CRP

CRP was analyzed in twelve studies (cohort N = 7, case–control N = 3, cross-sectional N = 2; mean ErasmusAGE score 4.3 out of 10.0), three of high quality and nine of low quality.

Preconception: Higher levels of serum or follicular fluid CRP in obese and overweight/obese women, compared to women of normal weight, were established in three studies, whereas one study of overweight/obese women showed no difference in serum levels [42, 44, 49]. Also, a positive correlation was observed between BMI and CRP levels in two studies, in which each 1kg/m^2^ increase in BMI was associated with 14% increase in follicular fluid CRP [49, 51].

First trimester: CRP was shown to be higher among obese women compared to lower BMI groups in five studies [70, 79–81, 87]. However, one study reported non-significantly higher levels of CRP among obese women [86]. Also, BMI was positively correlated with serum CRP levels in five studies [70, 79, 81, 82, 85]. In the Bodnar et al*.* study, a 5 unit increase in BMI was associated with 46% increase in serum CRP [81].

#### Interleukins (ILs)

The ILs were analyzed in nine studies of low quality and included IL-2, IL-4, IL-6, IL-8, IL-10, IL-18, IL-33, IL-1α and IL-1β (cohort N = 4, cross-sectional N = 3, case–control N = 2; mean ErasmusAGE 2.8 out of 10.0).

Preconception: Higher levels of follicular fluid IL-6 were observed in obese women compared to lower BMI groups in one study; whereas no difference was reported in the serum levels in two other studies [42, 49, 54]. BMI positively correlated with serum IL-18 levels in one study [53]. However, no effect of obesity was found on the levels of serum IL-1α, IL-1β, IL-2, IL-4, IL-8, IL-10 and IL-18, nor follicular fluid levels of IL-8 and IL-18 [49, 52, 54]. Also, BMI did not correlate with follicular fluid levels of IL-6, IL-10 or IL-18 in two studies [51, 53].

First trimester: Levels of IL-6 were higher among obese women compared to women of normal weight in one study [86]. In another study, BMI correlated negatively with IL-33 levels [35]. No significant difference was observed in serum levels of IL-1β, IL-8 and IL-10 between obese women compared to women of normal weight in any study [83, 86].

#### TNF-α

TNF-α was analyzed in five different studies of low quality (mean ErasmusAGE score 2.6 out of 10.0).

Preconception: No difference was observed in serum or follicular fluid TNF-α levels in obese and overweight/obese women compared to women of normal weight; and there was no correlation with BMI in three studies [42, 49, 51]. However, in one study, obesity class II women had higher TNF-α levels compared to lower BMI groups [54]. In another study, TNF-α levels correlated positively with fat percentage [42].

First trimester: No difference in TNF-α levels was observed between obese women and women of normal weight [86].

#### Other inflammatory biomarkers

Monocyte chemotactic factor-1 (MCP-1) was analyzed in three different studies of low quality (mean ErasmusAGE score 3.0 out of 10.0), whereas chemokine (C–C motif) ligand 2 (CCL2), chemokine (C-X-C motif) ligand 3 (CXCL3) and IL-34 genes, eotaxin, epidermal growth factor (EGF), fibroblast growth factor 21 (FGF21), glycoprotein acetylation A (GlycA), granulocyte macrophage-colony stimulating factor (GM-CSF), and soluble intercellular adhesion molecule-1 (sICAM-1) were analyzed only once (ErasmusAGE scores ≤ 5.0 out of 10.0).

Preconception: Ten genes involved in chemokine and cytokine pathways were differentially regulated between overweight/obese women and women of normal weight, with BMI correlating positively with CXCL3 and IL-34 gene expression [42]. One study showed higher serum and follicular fluid MCP-1 levels in obese women compared to women of normal weight and a positive correlation with BMI, whereas no correlation between follicular fluid MCP-1 and BMI was observed in another study [49, 51]. Serum levels of CCL2 were higher in overweight/obese women compared to women of normal weight, whereas no difference was observed in follicular fluid levels [42]. However, BMI correlated positively with GM-CSF levels [49]; although no difference was observed in the serum levels of GM-CSF, EGF or eotaxin between obese women compared to women of normal weight [49]. Also, BMI did not correlate with sICAM-1 levels in follicular fluid [51].

First trimester: Serum MCP-1 levels were higher by 42% in obese women compared to women of normal weight in one study [83]. Levels of GlycA were higher in obese women compared to overweight women and showed a positive association with BMI [70]. Also, FGF21 levels were positively correlated with BMI and fat mass [84].

##### Highlight

Obesity increases CRP levels thus exacerbating the inflammatory process across the periconceptional period.

### One-carbon metabolism biomarkers

One-carbon metabolism is a sequence of interlinked metabolic cycles providing one-carbon units for biosynthetic processes fundamental for cellular function [90]. Tables 2 and 3 summarize associations between maternal obesity and one-carbon metabolism biomarkers for the preconception period and first trimester, respectively.

#### Folate

Folate was analyzed in three studies (cohort N = 2, case–control N = 1; mean ErasmusAGE score 5.7 out of 10.0), one of high quality and two of low quality.

First trimester: Obese women had lower serum folate levels compared to women of normal weight in two studies, and higher odds for folate deficiency in one study, whereas no difference was observed in either serum or red blood cell (RBC) in two studies [80, 88, 89]. In one study, BMI negatively associated with serum folate levels whereas no association was observed with RBC folate [88].

#### Vitamin B12

Vitamin B12 was analyzed in two cohorts (mean ErasmusAGE score 6.5 out of 10.0), one of high and one of low quality.

First trimester: Obese women had lower levels of vitamin B12 compared to women of normal weight in two studies, and they revealed higher odds for vitamin B12 deficiency in one study [80, 88]. Also, BMI negatively associated with vitamin B12 levels in one study [88].

#### Methionine

Preconception: Methionine was analyzed in one study (ErasmusAGE score 2.0 out of 10.0) and showed higher levels in obese women compared to women of normal weight; and there was a positive correlation with BMI [50].

#### Folate transporters

First trimester: One study analyzed placental tissue (ErasmusAGE score 4.0 out of 10.0) and reported that obese women had lower mean expression of microvillus plasma membrane (MVM) reduced folate carrier (by 19%), MVM folate receptor alpha (by 17%) and methyl tetrahydrofolate uptake (by 52%) compared to women of normal weight [89]. However, no difference in expression levels were observed for MVM proton coupled folate transporter between obese women and women of normal weight [89].

##### **Highlight**

Obesity reduces folate and vitamin B12 levels in the first trimester of pregnancy which interferes with one-carbon metabolic pathways.

## Discussion

This systematic review addressed the impact of maternal obesity on several biomarkers of the endocrine, inflammatory and one-carbon metabolic pathways during the periconceptional period, extended to cover the first trimester of pregnancy. Outcomes were presented as a function of obesity, combined overweight/obesity or BMI. Throughout the periconceptional period, obesity was associated with a variety of biomarkers of the endocrine, inflammatory, as well as one-carbon metabolic pathways. Dysregulation in these three pathways, as a consequence of obesity, can lead to adverse maternal, fetal and offspring health outcomes. Here we focus on biomarkers relevant to obesity and pregnancy that were analyzed in multiple studies, as well as biomarkers from single studies of high quality based on ErasmusAGE scores.

### Endocrine biomarkers

#### Adipokines

##### Leptin

The present review demonstrated that levels of leptin are elevated in obese women and correlate positively with BMI at both systemic and local levels throughout the periconceptional period [35, 40, 42, 45, 50, 51, 62, 65, 66, 68, 71, 74, 76–78]. The results are consistent with other studies, confirming the effect of obesity on increasing leptin levels [91, 92]. Moreover, levels of leptin in follicular fluid are also positively correlated with serum levels in pregnant and non-pregnant women, suggesting a connection between serum and follicular fluid levels [45]. Leptin is directly secreted from the white adipose tissue and its levels are proportional to the level of adiposity [93, 94]. Circulating leptin acts on the brain to regulate energy homeostasis via its actions on satiety [95]. Leptin transport across the blood brain barrier is decreased and endoplasmic reticulum stress is increased in obese individuals, which may contribute to the development of leptin resistance, a pathological condition that induces hyperleptinemia [95]. Weight loss is associated with reduced adiposity, therefore decreasing leptin levels. This was demonstrated by a recent meta-analysis that showed that Orlistat use (a weight loss drug) was effective in decreasing leptin levels [96].

##### Adiponectin

Obesity increases CRP levels and is linked to mitochondrial dysfunction in adipose tissue leading to decreased adiponectin synthesis [97–99]. Moreover, obesity increases the expression of caveolin-1, a major component of the caveolae (small membrane invaginations), which attenuates leptin-dependent adiponectin secretion [100, 101]. This is consistent with results from the first trimester when obesity was related to reduced adiponectin levels; which would hinder its anti-inflammatory effect [65–67, 102]. Consistent with our findings, two previous studies demonstrated that obese women have low circulating adiponectin levels compared to non-obese women [103, 104]. Moreover, a decrease in body weight during a weight loss program was associated with increased serum adiponectin levels in overweight/obese women [105]. On the other hand, adiponectin concentrations in follicular fluid were not altered by obesity preconceptionally and were not related to serum levels. This suggests that regulatory mechanisms controlling adiponectin concentrations are different at systemic and local levels [106, 107].

#### Thyroid biomarkers

Thyroid hormones are involved in regulating body metabolism, yet it is unclear whether altered thyroid function is a cause or consequence of obesity [108]. In this review, obese women exhibited reduced FT4 levels; whereas levels of TSH were increased during first trimester [55–57, 61]. Similarly, TSH and FT4 levels in pregnant women were influenced by maternal weight [109]. Placental secretion of hCG contributes to increased FT4 levels in normal pregnancies whereas, in obese women, levels of hCG are decreased which diminishes the thyrotropic effect of hCG [110]. Subsequently, low FT4 levels reduce the negative feedback of FT4 on TSH secretion [111]. Moreover, higher Tg antibody positivity and iodine deficiency, associated with obesity, might contribute to the lower TT4 levels reported [55, 56]. However, no association between BMI and TSH levels at a mean of 16.6 weeks gestational age was observed [113]. This suggests that other factors, such as gestational age and iodine status, can influence the association between adiposity and thyroid hormone levels.

#### Steroids and gonadotropins

##### Estrogen, LH, FSH

Obesity did not alter estradiol and LH levels except in an Asian population of younger women [42, 43, 46, 48, 49, 52]. Results are consistent with other studies demonstrating the absence of effect [114, 115]. However, the effect of ethnicity requires further exploration [116]. Because estrogen was not influenced by obesity, its effect on FSH may be similar to that observed in women of normal weight, which was reported in the majority of studies analyzing FSH [42, 48, 52, 117]. Nevertheless, Buyuk et al. reported lower FSH levels with no alteration in estradiol [49]. However, the small sample size beholds the possibility of bias. Also, the purpose of ART treatment is to provide optimal conditions for successful ovulation, which might explain the absence of difference in sex hormones measured after a short time of hormonal treatment.

##### Progesterone

A negative relationship between obesity and progesterone levels was demonstrated during the first trimester which can in part be explained by the effect of leptin on reducing progesterone secretion [59, 64, 67, 118]. Also, lower hCG levels associated with obesity might contribute to low progesterone levels, as hCG supports the production of progesterone by the corpus luteum [119]. Lower progesterone levels were present in obese pregnant women with a male but not a female fetus [67]. Likewise, the association between progesterone levels and fetal steroid profile, timing of delivery and birth weight were also dependent on fetal sex [59, 120, 121]. During the preconception period, the effect of obesity on progesterone levels was only established in sub-fertile women undergoing ART treatment and in younger women, which suggests an additional effect of age and mode of conception on progesterone levels [41, 43].

##### HCG

HCG is secreted from trophoblasts, used for pregnancy testing and required for the maintenance of pregnancy [122]. During the preconception period, hCG is detectable in women undergoing ART or can originate from tumors [123, 124]. The levels of hCG in the periconceptional period, as a result of exogenous hCG treatment or pregnancy, were decreased in obese women and associated negatively with BMI [46, 47, 60, 63]. The pathophysiology of this relationship is unclear, but results are consistent with previous studies, supporting this negative relationship [125, 126]. By way of illustration, when obese women are injected with hCG, they have lower Cmax and hCG compared to women of normal weight [127].

#### Insulin

Metabolic alterations during pregnancy can lead to decreased insulin sensitivity which might be exacerbated in obese women [128, 129]. The associated increase in insulin levels and in insulin resistance with obesity was reported during the first trimester of pregnancy; observations comparable to other studies, emphasizing the risk of hyperinsulinemia among obese women [58, 65, 67, 69–71, 78, 130, 131]. During the preconception period, two out of four studies did not show an effect of obesity on insulin levels, however, they were of small sample size and did not adjust for any confounders [42, 48]. The mechanisms behind the impact of obesity on insulin levels can be related to a decrease in total and high affinity insulin receptors, thus requiring increased insulin secretion [132]. Moreover, CRP impairs insulin receptor substrate 1 (IRS-1) which might explain the state of insulin resistance in obesity due to elevated CRP levels [133].

#### Other endocrine biomarkers

Visfatin and resistin were analyzed only once at each time period and from different sources, with inconsistent outcomes, thus a relationship cannot be established, especially since the involved outcomes were from low quality score studies [50, 77]. Increased testosterone levels in the first trimester might be due to the increased expression of 17beta-hydroxysteroid dehydrogenase type 5 (involved in the conversion of androstenedione to testosterone) in obese individuals [134, 135]. Yet, no change in testosterone levels were reported for the preconception period, which suggests a possible role of pregnancy in modifying biomarker levels in obese women [134, 135]. However, outcomes were from small sample sizes, thus lacking power to establish clear relationships. The relationship between cortisol and obesity was dependent on emotional state demonstrating a potential role of stress on cortisol levels, which is consistent with other findings [72, 136–138]. Outcomes on the effect of obesity on AMH levels were from low quality score studies, thus they lacked power to establish a clear relationship with obesity. However, lower levels were previously reported in obese women [139, 140].

### Inflammatory biomarkers

#### CRP

Obesity is a chronic inflammatory state associated with elevated CRP levels, a recognized marker of systemic inflammation. This was evident in the present systematic review throughout the periconceptional period [70, 79–82, 85–87]. Outcomes are in line with a previous meta-analysis that showed an association between BMI and CRP levels in women [141]. Moreover, increased CRP levels were also reported in follicular fluid suggesting the activation of an inflammatory cascade in follicles of obese women [42, 44, 51]. This effect can be related to the role of adipose tissue inducing CRP gene expression in obese individuals [142]. For example, genes involved in chemokine and cytokine pathways were differentially expressed in the obese state [42]. Moreover, adipose tissue secretes IL-6, which can be elevated in obese individuals, promoting the release of CRP [54, 86, 143, 144].

#### Interleukins

There was no effects of obesity on the majority of interleukins studied during the periconceptional period [42, 49–54, 83, 86]. Outcomes for IL-6 were inconsistent, where two studies showed no effect preconceptionally, while one study reported elevated levels in obese individuals during the preconception period and first trimester [42, 49, 54, 86]. Increased serum IL-6 during the first trimester can be explained by the increased expression of IL-6 and IL-6 receptor in adipose tissue of obese women, and the effect of leptin in inducing IL-6 secretion by trophoblasts [118, 143]. Considering ILs, all studies were of low quality, thus limiting confidence in the effects of obesity, especially as the results contradict other studies in humans and rodents reporting a relationship with obesity [145–147].

#### TNF-α

TNF-α is implicated in the state of inflammation and metabolic complications associated with obesity [148]. From this review, the association between obesity and TNF-α levels during the periconceptional period remains controversial, with the majority of studies not showing a relationship [42, 49, 51, 86, 149]. Despite an absence of differences in TNF-α levels between overweight/obese and normal weight women, TNF-α levels correlated positively with fat mass which is a better indicator for adiposity [42]. Moreover, these studies were of low quality and those showing no relationship contradict others that showed increased levels, and adipose tissue expression, of TNF-α in obese individuals [42, 49, 51, 86, 150, 151].

#### Other inflammatory biomarkers

MCP-1 levels were elevated in obese women during the periconceptional period [49, 83]. Outcomes were from low quality score studies but comparable to others showing induced overexpression and levels of MCP-1 in obese individuals, suggesting an effect of obesity during the periconceptional period [152, 153]. In a mouse model, obesity was proposed to be a FGF21- resistant condition which might explain why its positive association with BMI was reported in the first trimester [84, 154]. Other inflammatory biomarkers such as CCL2, GM-CSF, EGF, sICAM-1, eotaxin and GlycA were analyzed once in low quality score studies, thus making it difficult to establish clear relationships.

### One-carbon metabolism biomarkers

Obesity was associated with lower vitamin B12 levels during the first trimester, whereas associations between obesity and folate levels were inconsistent; two studies showed decreased levels whereas one study, with a small sample size, demonstrated no association [80, 88, 89]. Low levels of folate and vitamin B12 perturb the functioning of one-carbon metabolism impairing DNA synthesis and chromatin methylation [4]. Previous studies reported a relationship between obesity and low folate and vitamin B12 levels; however, the cause-consequence relationship remains controversial [155, 156]. Inadequate dietary intake, altered distribution and absorption of micronutrients may contribute to the effect of obesity on lowering serum folate and vitamin B12 levels [157, 158]. On the other hand, folate deficiency and low vitamin B12 levels induce adipogenesis, which is attenuated with increased vitamin B12 and folate levels [159–161].

Obesity was associated with increased methionine levels in one low quality score study which had a small sample size [50]. This is not consistent with what would be expected due to the effect of obesity on reducing folate and vitamin B12 levels, both acting as cofactors in the remethylation of homocysteine to methionine, thus reducing methionine levels [4, 162].

### The endocrine, inflammatory and one-carbon metabolic axes

**Fig. 3:**
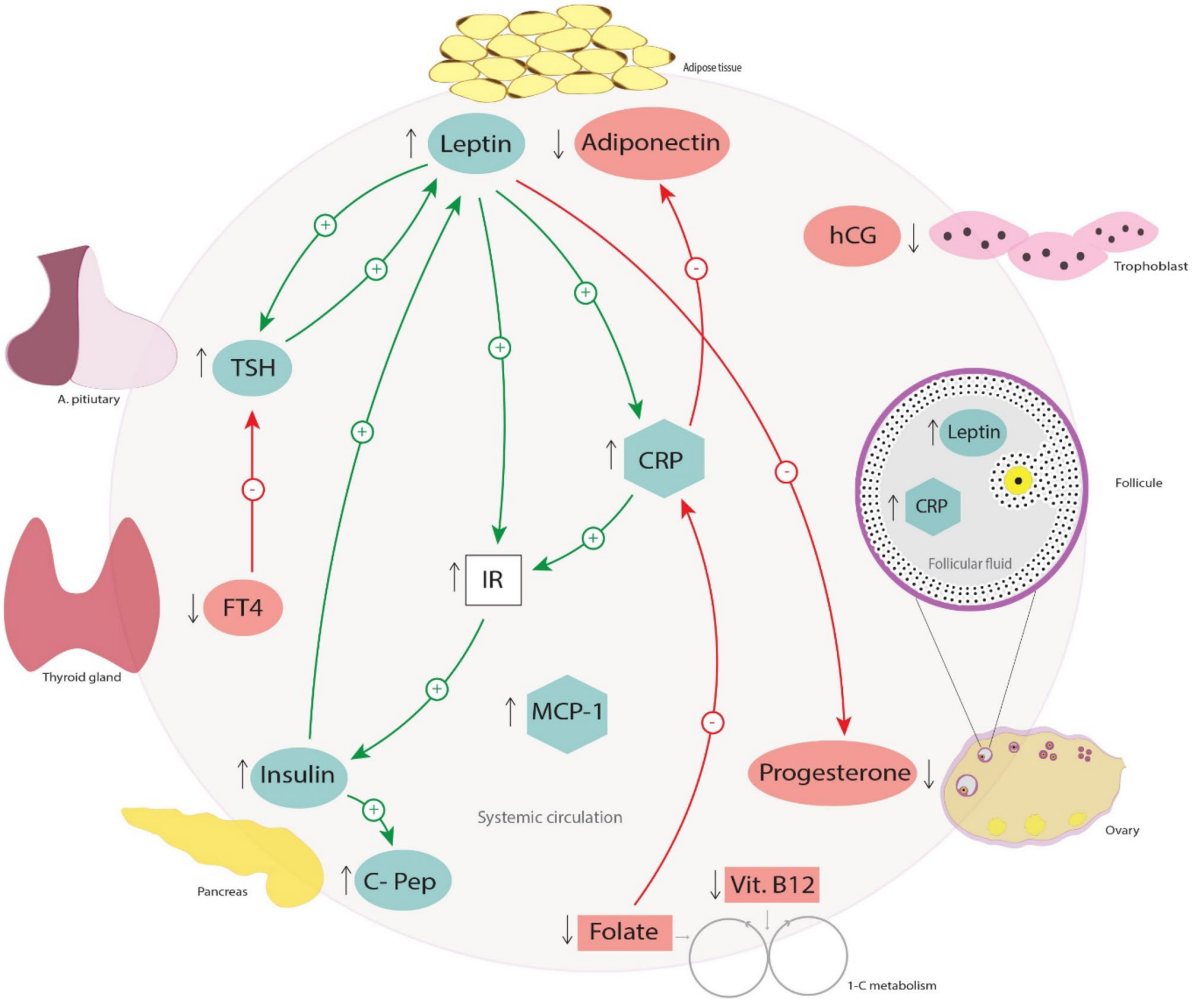
Major biomarkers of the endocrine, inflammatory and one-carbon metabolic pathways affected by maternal obesity, and possible connections throughout the periconceptional period based on human and animal studies. Levels of leptin, TSH, insulin, C-pep, MCP-1 and CRP are increased (↑) in obese women, whereas the levels of adiponectin, hCG, progesterone, vitamin B12, folate and FT4 are decreased (↓). FT4 inhibits (-) TSH secretion. TSH promotes (+) leptin secretion. Leptin promotes (+) TSH and CRP and inhibits (-) progesterone secretion. CRP and leptin increase (+) IR. IR increases (+) insulin levels. Insulin promotes (+) leptin secretion and increases (+) C-pep production. CRP inhibits (-) adiponectin production. Folate decreases (-) CRP production. Abbreviations: CRP, C-reactive protein; C-pep, C-peptide; FT4, free T4; IR; insulin resistance; MCP-1, monocyte chemoattractant protein-1; TSH, thyroid stimulating hormone; vit.; vitamin; 1-C, one-carbon

Figure 3 illustrates the possible connections between the major biomarkers of the endocrine, inflammatory and one-carbon metabolic pathways affected by obesity during the periconceptional period based on human and animal studies. As leptin is a potential biomarker of obesity, an interplay between leptin and other biomarkers can be considered. In trophoblast cells, the secretion of hCG and IL-6 was stimulated with the treatment and secretion of leptin [118, 163]. In turn, hCG has a stimulatory effect on leptin secretion and expression via the mitogen-activated protein kinase (MAPK) pathway [164, 165]. Yet, reduced levels were observed in obese individuals. In mice, obesity activates the MAPK pathway, suggesting that other biomarkers might also be involved through this pathway; stimulating leptin expression independent of hCG levels [165, 166]. In the obese state, leptin is elevated and this reduces insulin responsiveness and progesterone secretion [118, 167]. At the same time, insulin and cortisol promote the production of leptin in human adipocytes, indicating the possibility of crosstalk among biomarkers within the endocrine pathway of obese individuals [168]. Subsequently, elevated levels of C-peptide are related to elevated insulin levels as demonstrated in the present systematic review [50, 58]. The cause-consequence link between obesity and thyroid dysfunction is controversial. Low FT4 levels reduce negative feedback inhibition on TSH secretion. TSH stimulates leptin secretion, and hypothyroidism is linked to increased weight gain [169, 170]. Simultaneously, leptin induces TSH by stimulating expression of thyroid-releasing hormone [171]. In addition, leptin influences the inflammatory pathway through its actions on CRP [172]. On the other hand, CRP impairs IRS-1 and a positive association was found between insulin and CRP [133, 173]. Moreover, co-incubation of adipocytes with CRP reduced adiponectin gene expression [99]. On the other hand, a reduction in CRP levels is observed with folic acid supplement use, as well as improvement in adiponectin levels and insulin resistance with vitamin B12 supplement use [174–176].

### Dysregulation in endocrine, inflammatory and one-carbon metabolism biomarkers and clinical outcomes

**Fig. 4:**
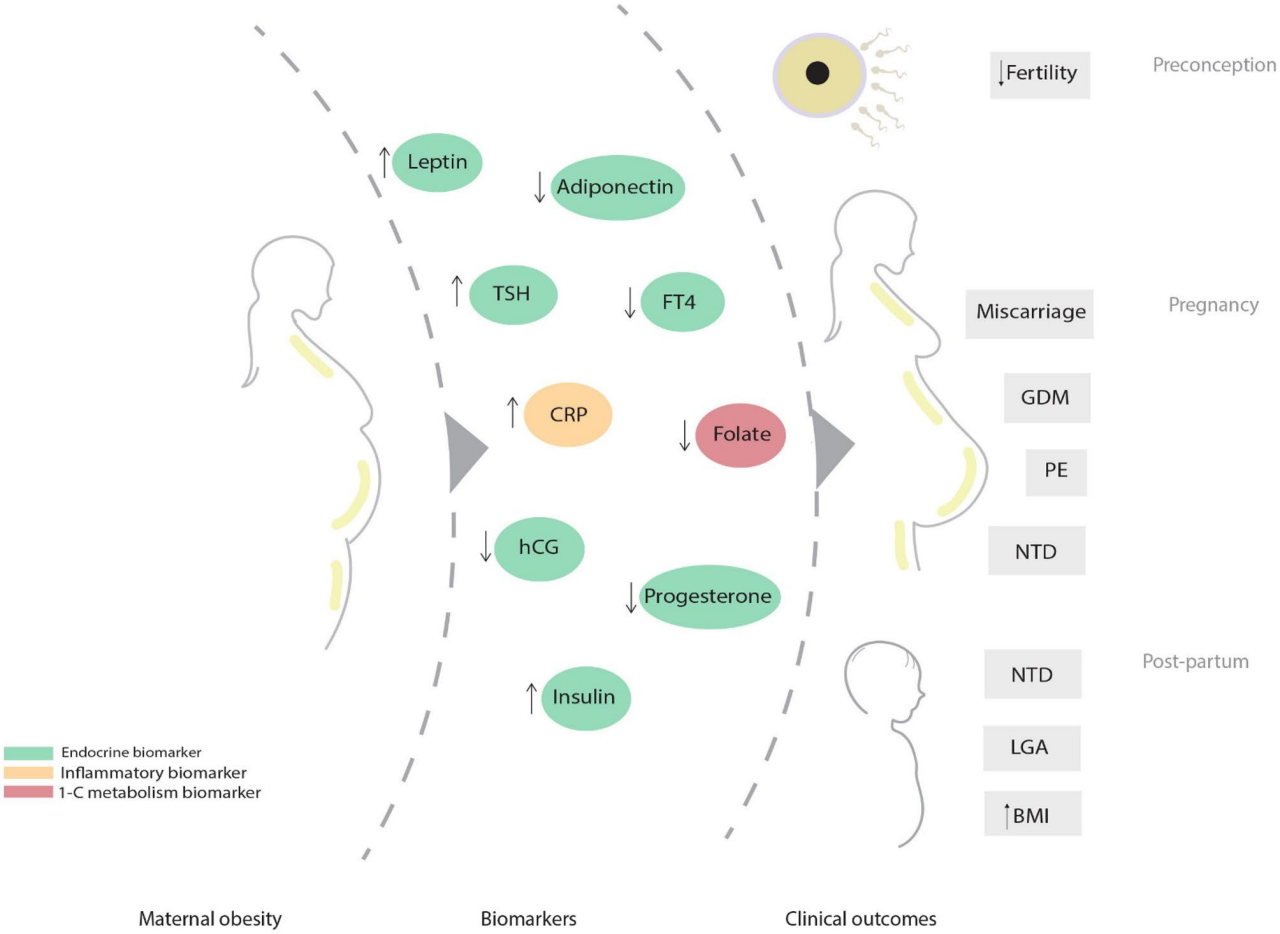
Hypothesized connection between maternal obesity, biomarkers of the endocrine, inflammatory and one-carbon metabolic pathways, and important clinical outcomes. Abbreviations: 1-C, one-carbon; BMI, body mass index; CRP, C-reactive protein; FT4, free T4; GDM, gestational diabetes mellitus; hCG, human chorionic gonadotropin; LGA, large for gestational age; NTD, neural tube defect; PE, preeclampsia; TSH, thyroid stimulating hormone. (↑) Increased levels. (↓) Decreased levels

Dysregulation in biomarker levels of the endocrine, inflammatory, and one-carbon metabolic pathways, as a result of maternal obesity, may impose adverse clinical outcomes related to fertility, pregnancy, and offspring health. Figure 4 illustrates the relationship between biomarkers of these three pathways and clinical outcomes. Obese women are at increased risk of subfertility which can be related to lower progesterone and increased leptin levels [40, 177]. Leptin can modulate reproductive function by affecting ovarian folliculogenesis and ovulation, and by perturbing the hypothalamic-pituitary-gonadal axis, such as by lowering progesterone and gonadotropin-releasing hormone levels [178, 179]. Subsequently, decreased progesterone levels can lead to changes in the endometrium physiology adversely affecting fertility, implantation and the maintenance of pregnancy [180]. Besides low progesterone levels, maternal obesity was also associated with lower HCG and higher TSH levels which can partly explain the increased risk of pregnancy loss among this population [181–187]. Low levels of HCG affect the uterine vasculature and placentation, whereas TSH is involved in endometrial physiology [188, 189]. During pregnancy, obese women showed increased insulin resistance, increased leptin and CRP levels, and decreased FT4 and adiponectin levels, which are associated with increased risk of cardio-metabolic pregnancy complications such as GDM and preeclampsia [34–37, 54, 81, 85, 173, 190, 191]. Endothelial dysfunction can be promoted by leptin, FT4, insulin and CRP while adiponectin improves endothelial dysfunction by inducing the production of nitric oxide [192–196]. Furthermore, leptin and CRP increase insulin resistance whereas adiponectin improves insulin sensitivity; primary features of GDM.

In terms of offspring health, high leptin levels are associated with increased infant BMI [197]. Similarly, increased insulin levels (mainly due to insulin resistance) and decreased adiponectin levels are correlated with increased risk of macrosomia and LGA newborns [191, 198–200]. Alterations in these biomarkers can influence placental nutrient transport, leading to a larger baby [201, 202]. Furthermore, placental and embryonic cerebellar growth can be influenced by the perturbations in one-carbon metabolism during the periconceptional period [203, 204]. Both folate and vitamin B12 deficiency can increase the risk of neural tube defects. Despite folic acid supplement use, obese women have an elevated risk of giving rise to children with neural tube defects, which can partly be explained by the lower expression of certain MVM placental folate transporters [89, 205].

### Strengths and limitations

This systematic review included a study population of obese women from different regions of the world supporting the general applicability of outcomes. Results are presented separately for associations between obesity, BMI or overweight/obesity and biomarkers; and between pre- and post-conception periods, permitting longitudinal interpretations and providing a clearer distinction of the origin of outcomes observed.

Limitations to be considered are, firstly, half of the studies (N = 25, 49%) did not report the tool for anthropometrics screening. Also, eight studies (14%) reported obesity based on self-reported anthropometrics. Though this tool is widely used in studies, the risk of self-reporting bias cannot be excluded [206, 207]. A second limitation relates to heterogeneity in time of reporting and measurement of body weight. Thus, outcomes were based on different BMI time-point measurements, although all were within the periconceptional period. Moreover, there was heterogeneity in the obese and control strata. For example, some studies included groups of combined overweight/obese women, which may interfere with interpreting the results solely attributable to obesity. With respect to the control group, some studies considered controls as non-obese or low BMI with BMI ranges transcending the known classifications, or they only had an overweight control group. Thirdly, several biomarkers analyzed for their relation to obesity or BMI were determined only on one occasion, thus making it difficult to interpret any dynamic effect. Also, some biomarkers were analyzed in low quality score studies, and/or in a small population, increasing the risk of a type II error. However, most observations were interpreted with support from high quality score studies for the same biomarker. Fourthly, the majority of included studies were not adjusted for covariates which are known to influence biomarker levels; particularly age and lifestyle factors [208, 209]. Finally, publication bias was not formally assessed, for example by using a funnel plot test, due to the different type of outcomes and few number of studies that analyzed each biomarker (N = 20, 46% of biomarkers were analyzed in one study; N = 15, 35% of biomarkers were analyzed in two to four studies; N = 5, 12% of biomarkers were analyzed in five to nine studies; N = 3, 7% of biomarkers were analyzed in at least ten studies).

### Implications for clinical practice and future research

This review suggests that maternal obesity is associated with altered periconceptional biomarker levels of the endocrine, inflammatory and one-carbon metabolic pathways which can affect fertilization, pregnancy, maternal and offspring health. The majority of these biomarkers were analyzed in clinical settings (i.e., by means of blood or follicular fluid sampling). Therefore, implementation into clinical practice could be considered appropriate for routine laboratory testing, as an early screening tool to identify and monitor the obese population at risk preconceptionally and shortly after conception, so within the periconceptional period. For example, analyzing and monitoring levels of leptin, adiponectin, FT4, insulin and CRP could assist in early identification of women at risk for preeclampsia or development of GDM. Identification of a high-risk population will allow for anticipatory obstetrical management including counseling for healthier lifestyle behaviour and more antenatal appointments. Besides their predictive value, these biomarkers can be also used for more personalized and effective management of adverse associated health risks later in life [210–212]. Of foremost importance, findings of this review support the known importance of weight loss to counteract the detrimental effects caused by obesity. This can be complemented by monitoring the reported biomarker levels. Furthermore, patient-tailored interventions (particularly those targeting lifestyle) to optimize health and biomarker levels among obese women, in general and more specifically the ones wanting to conceive, are recommended. For example, a change in dietary patterns and physical training were effective in reducing leptin, insulin and CRP levels, and increasing adiponectin levels in obese adults [213–215].

In light of these findings, additional research to identify the predictive value of the most promising biomarkers, particularly leptin, adiponectin, hCG, insulin, progesterone and CRP is recommended. Also, we encourage separate studies with obese and overweight individuals, and to have a normal BMI control group identified based on previously established criteria.

## Conclusion

Findings from this systematic review reveal that maternal obesity can alter levels of several biomarkers throughout the periconceptional period associated with disruption of the endocrine, inflammatory and one-carbon metabolic pathways. Importantly, maternal obesity was associated with higher leptin, insulin, TSH and CRP levels, and lower adiponectin, progesterone, FT4, hCG, folate and vitamin B12 levels. These biomarkers help to identify possible underlying pathophysiological mechanisms leading to adverse clinical outcomes. While the measurement of biomarkers is an applicable tool to potentially predict the risk of future adverse health outcomes, their clinical usefulness is still limited. Additional research on the predictive value of the optimal set of biomarkers is warranted for their use in clinical settings. Based on the current analysis, biomarkers of most interest include leptin, adiponectin, hCG, insulin, progesterone and CRP.

## Supplementary Information

Below is the link to the electronic supplementary material.

Supplementary file1 (DOCX 1.04 MB)

## Data Availability

Not applicable.

## Abbreviations

AMH: Anti-Müllerian hormone
ART: Assisted reproductive technology
BMI: Body mass index
CCL2: Chemokine (C–C motif) ligand 2
CRP: C-reactive protein
CXCL3: Chemokine (C-X-C motif) ligand 3
EGF: Epidermal growth factor
FGF21: Fibroblast growth factor 21
FSH: Follicle-stimulating hormone
FT4: Free T4
GDM: Gestational diabetes mellitus
GLP-1: Glucagon-like peptide 1
GlycA: Glycoprotein acetylation A
GM-CSF: Granulocyte macrophage-colony stimulating factor
HCG: Human chorionic gonadotropin
HOMA: Homeostatic model assessment
HOMA-IR: HOMA for insulin resistance
HOMA-IS: HOMA for insulin sensitivity
IGF-1: Insulin-like growth factor 1
IL: Interleukin
IRS-1: Insulin receptor substrate 1
LGA: Large for gestational age
LH: Luteinizing hormone
MCP-1: Monocyte chemoattractant protein-1
MVM: Microvillus plasma membrane
PRISMA: Preferred Reporting Items for Systematic Reviews and Meta-Analyses
PROSPERO: International Prospective Register of Systematic Reviews
SHBG: Sex hormone-binding globulin
SICAM-1: Soluble intercellular adhesion molecule-1
SOB-R: Soluble leptin receptor
Tg: Thyroglobulin
TNFα: Tumor necrosis factor alpha
TSH: Thyroid stimulating hormone
TT4: Total T4
WHO: World Health Organization
