# Supplementary material for: Periconceptional biomarkers for maternal obesity: a systematic review
Source: Rev Endocr Metab Disord. 2022 Dec 15;24(2):139–75. doi: 10.1007/s11154-022-09762-5 (PMC10023635; doi:10.1007/s11154-022-09762-5)
Supplement: Supplementary file 1 — Supplementary file1 (DOCX 1.04 MB) [file 11154_2022_9762_MOESM1_ESM.docx]

**Periconceptional biomarkers for maternal obesity: A systematic review**

Batoul Hojeij^1^, Melek Rousian^1^, Kevin D. Sinclair^2^, Andras Dinnyes^3,4,5^, Régine P.M. Steegers-Theunissen^1^ and Sam Schoenmakers^1^

^1^Department of Obstetrics and Gynecology, Erasmus MC, University Medical Center, Rotterdam, 3015GD, The Netherlands

^2^School of Biosciences, Sutton Bonnington Campus, University of Nottingham, Leicestershire, LE12 6HD, UK

^3^BioTalentum Ltd., Godollo, 2100, Hungary

^4^Department of Cell Biology and Molecular Medicine, University of Szeged, Szeged, 6720, Hungary

^5^Department of Physiology and Animal Health, Institute of Physiology and Animal Nutrition, Hungarian University of Agriculture and Life Sciences, Godollo, 2100, Hungary

Correspondence**:** Sam Schoenmakers

Email address: s.schoenmakers@erasmusmc.nl

Table S1: Literature search string of the systematic review.

| **Database** | **Search String** | **Number of records** |
| --- | --- | --- |
| Embase | ((('obesity'/de) AND ('pregnancy'/exp OR 'pregnant woman'/de OR 'mother'/de OR parity/de)) OR 'maternal obesity'/de OR ((mother* OR maternal* OR pregnan* OR gestion* OR first-trimester* OR gravid*) NEAR/6 (obes* OR overweight* OR adipos*)):ab,ti) AND ('biological marker'/de OR 'cytokine'/exp OR 'lipidomics'/exp OR 'endocrinology'/exp OR 'signal transduction'/exp OR 'hormone'/de OR 'blood level'/exp OR Cholesterol/exp OR 'folic acid'/exp OR 'carbon metabolism'/exp OR genetics/de OR 'epigenetics'/de OR heredity/de OR 'genetic association'/exp OR 'genetic association'/exp OR 'genetic heterogeneity'/exp OR 'genetic stability'/exp OR 'genome'/exp OR 'genotype'/exp OR 'mutation'/exp OR 'inheritance'/exp OR phenotype/exp OR 'DNA methylation'/de OR 'gene expression'/de OR 'gene repression'/exp OR 'gene silencing'/exp OR 'gene overexpression'/exp OR 'gene expression regulation'/exp OR transactivation/de OR 'protein induction'/de OR 'protein expression'/de OR metabolism/de OR 'hormone metabolism'/exp OR 'lipid metabolism'/exp OR 'energy metabolism'/exp OR 'mineral metabolism'/exp OR 'vitamin metabolism'/exp OR 'protein metabolism'/exp OR 'dyslipidemia'/de OR (biomarker* OR ((bio* OR blood OR serum OR serologic* OR inflammat* OR insulin-resistan* OR plasma*) NEAR/6 (marker* OR level* OR concentration*)) OR cytokine* OR lipidomic* OR endocrinolog* OR (signal* NEAR/3 (pathway* OR transduction*)) OR adiponectin* OR leptin* OR retinol-bind* OR resistin* OR hormon* OR Cholesterol OR lipoprotein* OR hdl OR ldl OR dyslipid* OR hypoadiponectin* OR folic-acid OR folate* OR metabolism* OR (metabolic* NEAR/3 factor*) OR hyperleptin* OR (lipid* NEAR/3 profile) OR tumor-necrosis-factor* OR C-reactive-protein* OR interleukin* OR jak-stat OR placenta*-growth-factor* OR heredit* OR genome* OR genotype* OR mutation* OR inheritan* OR phenotype* OR genotype* OR genetic* OR epigenetic* OR (DNA NEAR/3 methylat*) OR (protein* NEAR/3 (induc* OR expression*)) OR (gene* NEAR/3 (repressi* OR silenc OR overexpress* OR express*)) OR transactivat*):ab,ti) AND ('conception'/exp OR 'first trimester pregnancy'/de OR 'prepregnancy care'/de OR (concepti* OR periconcepti* OR preconcepti* OR prepregnan* OR pre-concepti* OR pre-pregnan* OR (first NEAR/3 trimester*) OR early-pregnan*):ab,ti) NOT ([Conference Abstract]/lim) AND [English]/lim | Initial search (until February 2020): N=1601  Updated search (February- December 2020): N=236 |
| Ovid Medline All | (((exp Obesity/) AND (Pregnancy/ OR Pregnant Women/ OR mothers/ OR parity/)) OR Obesity, Maternal/ OR ((mother* OR maternal* OR pregnan* OR gestion* OR first-trimester* OR gravid*) ADJ6 (obes* OR overweig7ht* OR adipos*)).ab,ti.) AND (Biomarkers/ OR Cytokines/ OR exp Lipidomics/ OR Endocrinology/ OR Signal Transduction/ OR Hormones/ OR Cholesterol/ OR Folic Acid/ OR Genetics/ OR Genetic Association Studies/ OR Epigenomics/ OR Heredity/ OR Genetic Heterogeneity/ OR Genome/ OR Genotype/ OR Mutation/ OR Phenotype/ OR DNA Methylation/ OR Gene Expression/ OR Gene Silencing/ OR Gene Expression Regulation/ OR Transcriptional Activation/ OR Metabolism/ OR Lipid Metabolism/ OR Energy Metabolism/ OR "Metabolic Networks and Pathways"/ OR Dyslipidemias/ OR (biomarker* OR ((bio* OR blood OR serum OR serologic* OR inflammat* OR insulin-resistan* OR plasma*) ADJ6 (marker* OR level* OR concentration*)) OR cytokine* OR lipidomic* OR endocrinolog* OR (signal* ADJ3 (pathway* OR transduction*)) OR adiponectin* OR leptin* OR retinol-bind* OR resistin* OR hormon* OR Cholesterol OR lipoprotein* OR hdl OR ldl OR dyslipid* OR hypoadiponectin* OR folic-acid OR folate* OR metabolism* OR (metabolic* ADJ3 factor*) OR hyperleptin* OR (lipid* ADJ3 profile) OR tumor-necrosis-factor* OR C-reactive-protein* OR interleukin* OR jak-stat OR placenta*-growth-factor* OR heredit* OR genome* OR genotype* OR mutation* OR inheritan* OR phenotype* OR genotype* OR genetic* OR epigenetic* OR (DNA ADJ3 methylat*) OR (protein* ADJ3 (induc* OR expression*)) OR (gene* ADJ3 (repressi* OR silenc OR overexpress* OR express*)) OR transactivat*).ab,ti.) AND (Fertilization/ OR Pregnancy Trimester, First/ OR Preconception Care/ OR (concepti* OR periconcepti* OR preconcepti* OR prepregnan* OR pre-concepti* OR pre-pregnan* OR (first ADJ3 trimester*) OR early-pregnan*).ab,ti.) NOT (news OR congres* OR abstract* OR book* OR chapter* OR dissertation abstract*).pt. AND english.la. | Initial search (until February 2020): N=178  Updated search (February- December 2020): N=25 |
| Web of Science Core Collection | AB=((((mother* OR maternal* OR pregnan* OR gestion* OR first-trimester* OR gravid*) NEAR/5 (obes* OR overweight* OR adipos*))) AND ((biomarker* OR ((bio* OR blood OR serum OR serologic* OR inflammat* OR insulin-resistan* OR plasma*) NEAR/5 (marker* OR level* OR concentration*)) OR cytokine* OR lipidomic* OR endocrinolog* OR (signal* NEAR/2 (pathway* OR transduction*)) OR adiponectin* OR leptin* OR retinol-bind* OR resistin* OR hormon* OR Cholesterol OR lipoprotein* OR hdl OR ldl OR dyslipid* OR hypoadiponectin* OR folic-acid OR folate* OR metabolism* OR (metabolic* NEAR/2 factor*) OR hyperleptin* OR (lipid* NEAR/2 profile) OR tumor-necrosis-factor* OR C-reactive-protein* OR interleukin* OR jak-stat OR placenta*-growth-factor* OR heredit* OR genome* OR genotype* OR mutation* OR inheritan* OR phenotype* OR genotype* OR genetic* OR epigenetic* OR (DNA NEAR/2 methylat*) OR (protein* NEAR/2 (induc* OR expression*)) OR (gene* NEAR/2 (repressi* OR silenc OR overexpress* OR express*)) OR transactivat*)) AND ((concepti* OR periconcepti* OR preconcepti* OR prepregnan* OR pre-concepti* OR pre-pregnan* OR (first NEAR/2 trimester*) OR early-pregnan*))) AND DT=(article) AND LA=(english) | Initial search (until February 2020): N=7  Updated search (February- December 2020): N=1 |
| Cochrane CENTRAL register of trials | (((mother* OR maternal* OR pregnan* OR gestion* OR first-trimester* OR gravid*) NEAR/6 (obes* OR overweight* OR adipos*)):ab,ti) AND ((biomarker* OR ((bio* OR blood OR serum OR serologic* OR inflammat* OR insulin-resistan* OR plasma*) NEAR/6 (marker* OR level* OR concentration*)) OR cytokine* OR lipidomic* OR endocrinolog* OR (signal* NEAR/3 (pathway* OR transduction*)) OR adiponectin* OR leptin* OR retinol-bind* OR resistin* OR hormon* OR Cholesterol OR lipoprotein* OR hdl OR ldl OR dyslipid* OR hypoadiponectin* OR folic-acid OR folate* OR metabolism* OR (metabolic* NEAR/3 factor*) OR hyperleptin* OR (lipid* NEAR/3 profile) OR tumor-necrosis-factor* OR C-reactive-protein* OR interleukin* OR jak-stat OR placenta* NEXT growth-factor* OR heredit* OR genome* OR genotype* OR mutation* OR inheritan* OR phenotype* OR genotype* OR genetic* OR epigenetic* OR (DNA NEAR/3 methylat*) OR (protein* NEAR/3 (induc* OR expression*)) OR (gene* NEAR/3 (repressi* OR silenc OR overexpress* OR express*)) OR transactivat*):ab,ti) AND ((concepti* OR periconcepti* OR preconcepti* OR prepregnan* OR pre-concepti* OR pre-pregnan* OR (first NEAR/3 trimester*) OR early-pregnan*):ab,ti) | Initial search (until February 2020): N=25  Updated search (February- December 2020): N=29 |

Table S2: ErasmusAGE^a^ quality score for systematic reviews adjusted for “Periconceptional biomarkers for Maternal Obesity: A systematic review”.

| **Item** | **Score** | **Description** | **Adjustments** |
| --- | --- | --- | --- |
| Study design | 0 | Studies with cross-sectional data collection | - |
|  | 1 | Studies with longitudinal data collection (both in retrospect and prospect) | - |
|  | 2 | Intervention studies | - |
| Study size (predefined)* | 0 | Small population for analysis | <100 |
|  | 1 | Intermediate population for analysis | 100-500 |
|  | 2 | Large population for analysis | >500 |
| Exposure |  | **Observational studies** |  |
|  | 0 | If the study used no appropriate exposure measurement method or if not reported | Tool for height and weight screening not specified |
|  | 1 | If the study used moderate quality exposure measurement methods | Tool for height or tool for weight screening specified |
|  | 2 | If the study used adequate exposure measurement methods | Weight and height measured or self-reported |
|  |  | **Intervention studies** |  |
|  | 0 | If the intervention was not described or not blinded | n/a |
|  | 1 | If the intervention was adequately single blinded | n/a |
|  | 2 | If the intervention was adequately double-blinded | n/a |
| Outcome | 0 | If the study used no appropriate outcome measurement method or if not reported | Analysis tools for biomarker measurement not specified |
|  | 1 | If the study used moderate quality outcome measurement methods | Analysis tools not specified for all biomarkers |
|  | 2 | If the study used adequate outcome measurement methods (Semen quality according to WHO, seminal epigenetics, miscarriage, preterm birth, small for gestational age, fetal abnormalities) | Analysis specified for all biomarkers |
| Adjustments ^b,c^ | 0 | If findings are not controlled for at least key confounders | No adjustments or stratification |
|  | 1 | If findings are controlled for key confounders | Age |
|  | 2 | If findings are additionally controlled for additional covariates or when an intervention is adequately randomized | Additional confounders |

^a^ Original: ErasmusAGE, 24 June 2013

This quality score can be used to assess the quality of studies included in systematic reviews and meta-analyses and is applicable to both interventional and observational studies. The score was designed based on previously published scoring systems (Carter et al., 2010 and the Quality Assessment Tool for Quantitative Studies). The quality score is composed of 5 items, and each item is allocated 0, 1 or 2 points. This allows a total score between 0 and 10 points, 10 representing the highest quality.

The version presented below is a general version and needs to be adapted for each review separately, e.g. concerning what study size is large or small within the study field, what exposure and outcome measurement methods are adequate, and what the key confounders are. Decisions on these detailed criteria should be based on literature, guidelines and/or discussions with experts. The criteria should be defined before the review process.

^b^ Needs to be specified for each review, based on literature, guidelines and/or expert opinions in the field.

^c^ Either adjusted for in the statistical analyses; stratified for in the analyses; or not applicable (e.g. a study in women only does not require controlling for sex)

Table S3: General characteristics and quality score of studies.

| **Author, year** | **Period of biomarker sampling** | **Period of BMI measurement** | **Population BMI (kg/m^2^)** | **Study design** | **Biomarker** | **QS** | **Pathw.** |
| --- | --- | --- | --- | --- | --- | --- | --- |
| C. Han *et al.*  (2015) | 4-8 GW | 4-8 GW | n/a | Cross-sectional | TSH and FT4 | 8 | EC |
| L. Mosso *et al.* (2016) | ≤ 14 GW | ≤14 GW | 26.3 ± 5.1 | Prospective observational cohort | TSH, TT4, FT4 | 8 | EC |
| V.J. Pop *et al.* (2013) | 12 GW | 8 GW | n/a | Prospective observational cohort | TSH and FT4 | 7 | EC |
| T.C. Plowden *et al.* (2019) | Days 2-4 of MC (for up to 6 consecutive cycles until pregnancy) | Preconception | 26 ± 6.4 | Prospective observational cohort | Leptin | 7 | EC |
| J. Bandres-Meriz *et al.* (2020) | 4-12 GW | 4-12 GW | Obesity  32.3 Normal /underweight  21.3 | Cross-sectional | Leptin, insulin, c-peptide | 7 | EC |
| A. Diemert *et al.* (2017) | 12-14 GW | First trimester | n/a | Prospective observational cohort | Progesterone | 6 | EC |
| A. Eskild *et al.* (2012) | Day 12 after ET | Within 6 month of ART | 23.7 ± 4 | Cross-sectional | hCG | 6 | EC |
| S. Gowachirapant *et al.* (2013) | 11 GW | Preconception | 20.9 | Cross-sectional | TSH, FT4, Tg | 6 | EC |
| C. Fattah *et al.* (2010) | 11.1 ± 1.6 GW | First trimester | 25.4 ±5.1 | Cross-sectional | Leptin | 6 | EC |
| P.C. Brady *et al.* (2018) | 9-13 days after blastocyst transfer or 11-15 days after ET cleavage stage | Preconception | n/a | Retrospective observational cohort | hCG | 5 | EC |
| J.Y. Goh *et al.* (2016) | 5-12 GW | First trimester | 23.0 ± 4.2 | Prospective observational cohort | Progesterone | 5 | EC |
| U. Andersson-Hall *et al.* (2020) | 8-12 GW | 8-12 GW | Obesity  34.5 ± 3.2 Healthy  22.4 ± 1.7 | Prospective observational cohort | Leptin, insulin, sOB-R, adiponectin | 5 | EC |
| A. Jara *et al.* (2020) | 12.4 ​± ​1.6 GW | Preconception | 26.8 ± 5.7 | Prospective observational cohort | Leptin, adiponectin | 5 | EC |
| H.M. Zeron *et al.*  (2012) | 10-12 GW | Preconception | Overweight /obesity  30.6  Normal  21.7 | Prospective observational cohort | Leptin | 5 | EC |
| M. Maliqueo *et al.* (2017) | First trimester | 6-8 GW | Obesity  33.4 Normal  22.9 | Prospective observational cohort | Insulin, adiponectin, progesterone, testosterone, SHBG, ASD, estradiol, estrone | 5 | EC |
| E. Petrella *et al.* (2014) | 9-12 GW | 9-12 GW | Overweight /obesity  32.2 ± 6.2 Normal  21.7 ± 2.2 | Prospective intervention cohort | Insulin | 5 | EC |
| P.C. Brady *et al.*  (2014) | Day of ET | preconception | 23.1 ± 3.2 | Retrospective observational cohort | Progesterone | 5 | EC |
| M.L. Ruebel (2017) | Oocyte retrieval | Preconception | Overweight /obesity  32.3 ± 1.8  Normal  22.3 ± 0.6 | Case-control | Insulin, leptin, TNF-α, CCL2, CRP, IL-6, FSH, LH, estradiol, genes | 4 | EC /Inf |
| N. Malti *et al.* (2020) | First trimester | Preconception | Obesity  34.5 ± 2.25 Normal  22 ± 2.5 | Prospective observational cohort | Leptin and insulin | 4 | EC |
| J.W. Luiza *et al.* (2015) | 6-16 GW | Preconception | Depressed 31.5 ± 7.7  Non-depressed 30.3 ± 7.4 | Case-control | Cortisol | 4 | EC |
| N. Houttu *et al.* (2018) | 13.2 ± 2.5 GW | Preconception | Obesity  34 ± 4  Overweight  27 ± 2 | Cross-sectional | Insulin, CRP, GlycA | 4 | EC /Inf |
| M.A Hamza *et al.* (2019) | First trimester | Preconception | Obesity  28.59 ± 2.49 Lean  21.9 ± 1.8 | Cross-sectional | Betatrophin | 4 | EC |
| L.T. Wang *et al.* (2020) | hCG day | Preconception | n/a | Cross-sectional | Estradiol, progesterone, LH | 4 | EC |
| H.M. Zeron *et al.* (2013) | 10-12 GW | Preconception | Overweight /obesity  15-43  Normal  16.8-24.6 | Prospective observational cohort | Leptin, adiponectin | 4 | EC |
| S.D. Valckx *et al.* (2012) | Oocyte retrieval | Preconception | n/a | Prospective observational cohort | Insulin, CRP, IGF-1 | 4 | EC /Inf |
| M.J. Hill *et al.* (2007) | Day of GnRH dose decrease | Preconception | 27.2 ± 5.2 | Prospective observational cohort | Leptin | 3 | EC |
| F. Beneventi *et al.* (2019) | 11-13 GW | First trimester | n/a | Case-control | Leptin, IL-33 | 3 | EC /Inf |
| S. Nelson *et al.* (2010) | 8-14 GW | 8-14 GW | 18.5-46 | Prospective observational cohort | AMH | 3 | EC |
| M. Shaarawy *et al.* (1999) | 6-11 GW | First trimester | 18.3 - 35.2 | Cross-sectional | Leptin | 3 | EC |
| M. Suto *et al.* (2019) | 10-14 GW | Preconception | Overweight /obesity  28.1 ± 3.5 Normal  21.2 ± 1.7 Lean  17.7 ± 0.7 | Prospective observational cohort | Leptin, adiponectin, visfatin, resistin | 3 | EC |
| D.T. Carrell *et al.* (2001) | 36 h after hCG injection | Preconception | High  34.0 ± 0.62 Medium  23.7 ± 0.20  Low  19.0 ± 0.13 | Cross-sectional | hCG, estradiol | 3 | EC |
| R. Matorras *et al.* (2012) | Oocyte retrieval | Preconception | n/a | Cross-sectional | hCG | 3 | EC |
| M. Peigné *et al.* (2020) | AMH: 2-3 days before oocyte retrieval  Other: 2-5 days of cycle | Preconception | Obesity  33.7  Normal  20.7 | Prospective observational cohort | AMH, testosterone, ASD, estradiol, LH, FSH, SHBG, insulin | 2 | EC |
| L. Lassance *et al.* (2015) | 7-12 GW | Preconception | Obesity  35.2 ± 6.5  Normal  21.2 ± 1.9 | Prospective observational cohort | Insulin, leptin, placental insulin regulated genes | 2 | EC |
| E. Buyuk *et al.* (2017) | Day 2 or 3 of MC before COH | Preconception | Obesity (S)  34.0 ± 3.8  Normal (S)  21.9 ± 1.3  Obesity (FF)  35.3 ± 4.8  Normal (FF)  22.2 ± 1.2 | Prospective observational cohort | CRP, MCP-1, IL-1α, IL-1β, IL-2, IL-4, IL-6, IL-8, IL-10, EGF, GM-CSF, TNF-α, eotaxin, FSH, estradiol | 2 | EC /Inf |
| M.B. Gonzalez *et al.* (2018) | Oocyte pickup | Preconception | 19.9-48.3 | Cross-sectional | Adiponectin, CRP, IL-6, IL-10, Leptin, MCP-1, sICAM-1 and TNF-α | 2 | EC /Inf |
| S. Kilic *et al.* (2009) | Oocyte pickup | Preconception | n/a | Cross-sectional | LH, FSH, IL-18 | 2 | EC /Inf |
| L. Bou Nemer *et al.* (2019) | Oocyte retrieval | Preconception | Obesity  37.4 ± 0.78  Overweight  26.7 ± 0.38  Normal  21.2 ± 0.84 | Prospective observational cohort | Insulin, methionine, gastric inhibitory polypeptide (GIP), glucagon, visfatin, ghrelin, GLP-1, resistin, c-peptide, adiponectin, leptin | 2 | EC /Inf /1-CM |
| T. Jaaskelainen *et al.* (2019) | First trimester | Preconception | PE  25.3 ± 5.2  Control 24.1 ± 4.4 | Prospective observational cohort | CRP | 9 | Inf |
| J.M. Scholing *et al.* (2018) | 13.5 ± 3.3 GW | Preconception | Total  22.9 ± 3.8  Obesity  33.8 ± 4 | Prospective observational cohort | Folate, vitamin B12, CRP | 8 | Inf /1-CM |
| L. Bodnar *et al.* (2005) | PE cases  13.6  ± 4.4 GW Controls  13.8  ± 4.1 GW | Preconception | n/a | Case-control | CRP | 7 | Inf |
| P.J. Maguire *et al.* (2015) | 12.5 GW | 12.5 GW | 17.4 - 40.9 | Retrospective observational cohort | CRP | 5 | Inf |
| L. Polari *et al.* (2018) | First trimester | Preconception | n/a | Prospective observational cohort | IL-1β, IL-10, and MCP-1 | 5 | Inf |
| E.F. Sutton *et al.* (2018) | <14 GW | <14 GW | 32.8 ± 5.4 | Prospective observational cohort | FGF21 | 5 | Inf |
| M. Wolf *et al.* (2001) | 11 ± 2 GW | n/a | PE  29 ± 8  Controls  23 ± 4 | Prospective observational cohort | CRP | 5 | Inf |
| L.M. Christian *et al.* (2014) | 11 ± 2.3 GW | First trimester | n/a | Prospective observational cohort | IL-6, TNF-α, IL-1β, IL-8, and CRP | 3 | Inf |
| A.J. Shhaeat *et al.* (2019) | First trimester | n/a | n/a | Case-control | CRP | 2 | Inf |
| V. Gunther *et al.* (2016) | Oocyte pickup | Preconception | n/a | Prospective observational cohort | IL-18 | 2 | Inf |
| S. La Vignera *et al.* (2011) | Ovarian pick up | Preconception | n/a | Cross-sectional | TNF-α, IL-6, IL-8 | 2 | Inf |
| E.G. O'Malley *et al.* (2018) | 12.1 ± 1.6 GW | 12.1 ± 1.6 GW | n/a | Prospective observational cohort | Folate, vitamin B12 | 5 | 1-CM |
| R.H. Jessel *et al.* (2020) | Obesity  14.6 ± 0.4 GW Normal  14.8 ± 1.2 GW | 8-22 GW | Obesity  33.6 ± 0.88  Normal  21.9 ± 0.51 | Case-control | MVM RFC, MVM PCFT, MVM FR-α, MTHF, folate | 4 | 1-CM |

Abbreviations: 1-CM, one-carbon metabolism; AMH, anti mullerian hormone; ASD, androstenedione; CCL2, chemokine (C-C motif) ligand 2; COH, controlled ovarian hyperstimulation; CRP, C-reactive protein; EC, endocrine; EGF, epidermal growth factor; ET, embryo transfer; FF, follicular fluid; FGF21, fibroblast growth factor 21; FR-α, folate receptor α; FSH, follicle stimulating hormone; GIP, gastric inhibitory polypepetide; GM-CSF, granulocyte macrophage-colony stimulating factor; GlycA, glycoprotein acetylation; GW, gestational weeks; h, hour; hCG, human ghorionic gonadotropin; IGF-1, insulin-like growth factor 1; IL, interleukin; LH, luteinizing hormone; Inf, inflammatory; MC, menstrual cycle; MCP-1, monocyte chemotactic protein-1; MTHF, methyltetrahydrofolate; MVM, microvillous plasma membrane; n/a, not available; pathw., pathway; PCFT, proton coupled folate transporter; PE, preeclemptic; QS, quality score; RBC, red blood cells; RFC, reduced folate carrier; S, serum; SHBG, sex hormone-binding globulin; sICAM-1, soluble intercellular adhesion molecule-1; SOB-R, soluble leptin receptor; Tg, thyroglobulin; TNF, tumor necrosis factor; TSH, thyroid stimulating hormone.


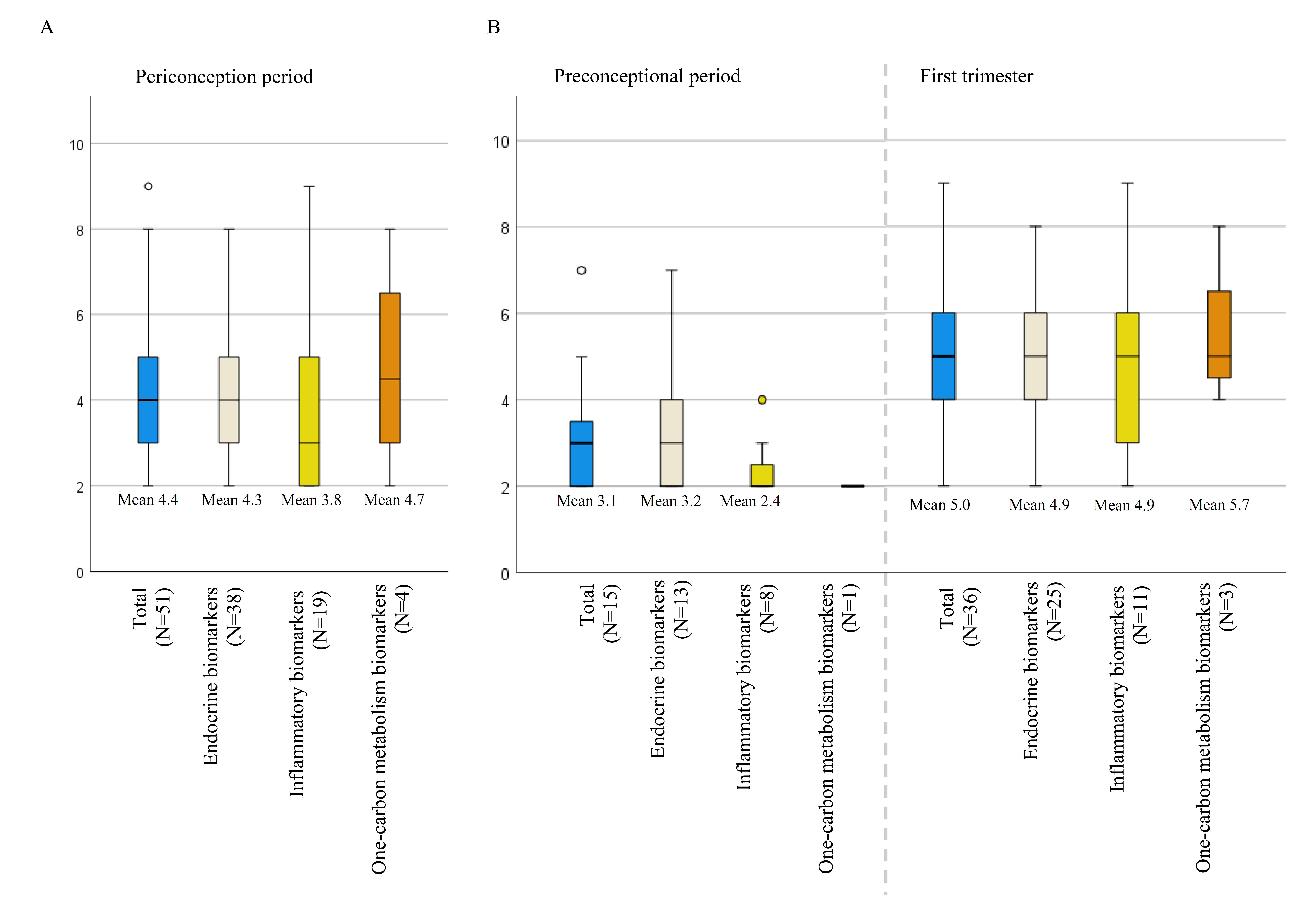


**Fig. S1** Boxplots of ErasmusAGE scores of all included articles in the systematic review for the periconceptional period (A), preconceptional period (B) and first trimester (B). The periconception period represents the preconceptional period and first trimester.
